# Alternative beliefs in psychedelic drug users

**DOI:** 10.1038/s41598-023-42444-z

**Published:** 2023-09-30

**Authors:** Alexander V. Lebedev, Kasim Acar, Otilia Horntvedt, Andrés E. Cabrera, Otto Simonsson, Walter Osika, Martin Ingvar, Predrag Petrovic

**Affiliations:** 1 Department of Clinical Neuroscience, Karolinska Institutet, Stockholm, Sweden; 2 Center for Cognitive and Computational Neuroscience, Karolinska Institutet, Stockholm, Sweden; 3 Department of Clinical Neuroscience, Center for Psychiatry Research, Karolinska Institute, Stockholm, Sweden

**Keywords:** Epidemiology, Addiction, Human behaviour

## Abstract

Previous research has suggested that classical psychedelics can foster significant and enduring changes in personality traits and subjective wellbeing. Despite the lack of evidence for adverse effects on mental health stemming from psychedelic use, concerns persist regarding the capacity of these substances to modulate information processing and attitudes towards factual data. The aim of the present study was to investigate the propensity for accepting alternative facts and the general treatment of knowledge within a sample of 392 participants, 233 of whom reported at least a single incidence of psychedelic use in their lifetime. To do this, we leveraged step-wise methods of linear modelling investigating effects of demographics, psychiatric conditions and concomitant drug use. Our findings revealed a moderate positive association between psychedelic use and beliefs in alternative facts, as well as the specific belief that facts are politically influenced. However, no links were found for favouring intuition over evidence when confirming facts. Among other investigated drugs, only alcohol was negatively associated with beliefs in alternative facts. Taken together, our results support the link between psychedelic use and non-conformist thinking styles, which can be attributed to the psychological effects of the drugs themselves, but may also mirror a common trait related to unconventional beliefs and illicit substance use.

## Introduction

Over the last decade, several nations have initiated policies to liberalize drug use. Following cannabis, classical psychedelics such as LSD, psilocybin, and DMT are the group of substances most impacted by these reforms. These changes have been partially inspired by the effect of these substances on mental health and wellbeing, as well as by evidence from several small-scale clinical trials showcasing their medicinal utility. Notably, these substances have been proposed as potential treatments for a variety of psychiatric conditions including posttraumatic stress disorder, depression, anxiety, and certain addictions^1–6^. The current landscape calls for further studies focusing on the previously under-explored impacts of psychedelics to better comprehend their recreational and medicinal contexts.

Beyond the scarcity of data related to possible adverse effects, the impact of psychedelics on several psychological aspects has not been meticulously studied. For instance, while there exists a substantial corpus of research exploring the relationship between drugs and personality structure^7–9^, few studies have probed how these substances might influence individuals' belief systems^10^. Nevertheless, it has been hypothesized that the effects of certain substances, like psychedelics, may catalyse changes in life attitudes^11^, as well as political and environmental views^12,13^. This premise, however, has been challenged by more recent studies conducted with larger samples^14^. In the wake of these findings, some researchers posit that the eventual prohibition of psychedelic drugs was, to a degree, politically motivated^15^. An example of this is the concern that the non-conformist perspectives and conspiracy ideation emerging from the rising counter-culture linked with psychedelic use might negatively impact society^16^.

Beliefs in alternative facts and conspiracy theories span a broad spectrum of themes with diverse magnitudes of impact on individual and societal wellbeing. Some can potentially be detrimental to physical and mental health^17–20^, some may pose social threats^21,22^, whilst other conspiracy ideas may be more neutral to the societal and individual functioning^23^. Moreover, some contend that certain conspiracy ideation can be seen as healthy scepticism or a way of challenging societal and ideological norms^24^. Others contend that there are significant differences that distinguish conspiracy theories from the hidden political agendas that happen behind the scenes of most governments^25^. While there exists a considerable body of research on pathological conspiracy ideation, it's crucial to remember that to date, there are no universally accepted tools to differentiate between authentic conspiracy theorists and so-called “healthy sceptics” within the general population.

Considerable effort has been made to examine the associations between the use of various drugs and personality structure, which are foundational for the formation of diverse belief systems. An interdisciplinary group of researchers, for instance, identified significant differences in personality profiles between drug users and non-drug users^8^. In addition to certain commonalities, some personality traits appeared to be drug-specific. Alcohol use and abuse, for instance, has been associated with high levels of extraversion^7,9^. This aligns with the perception of alcohol as a “social lubricant” that diminishes defences, engenders trust, and thus enhances sociability. This perspective was corroborated in a study assessing the impacts of alcohol consumption on social interactions and generalized trust, which were both found to be positively correlated with alcohol consumption^26^. Besides fostering interpersonal relationships on an individual level, generalized trust can be regarded as an integral component of social capital, a concept that also encompasses social networks, norms, and support^26,27^. According to social capital theory, trust acts as the adhesive binding society together and is associated with faith in political institutions and satisfaction with democracy^27,28^. Interestingly, social capital, in the form of community-level participation has also been linked to alcohol consumption^29,30^. This illustrates how alcohol, relative to other drugs, may be viewed as normative and might contribute to promoting more conventional viewpoints as opposed to non-conformist ones.

Along with nicotine and cannabis, psychedelic drug use, on the other hand, has been linked to *openness*^8,9,31,32^, a personality trait tied to non-conformity and liberalism^33,34^. In most population studies probing these links, it is challenging, if not impossible, to deduce causal relationships between personality structure and patterns of drug use; that is, whether a drug induces a personality change or whether a particular personality structure predisposes an individual to prefer a certain type of drug. However, this association has been investigated more thoroughly for classical psychedelics in a series of experimental studies demonstrating enduring increases in personality trait openness following a single psychedelic experience^35–37^. The same has been observed for nature-relatedness^12,13^ and, to a less conclusive degree, for shifts in political views^13,14^.

While these findings are indeed significant, there remains a gap in the literature regarding the relationship between the use of psychedelic drugs, non-conformist thinking, and the general approach to knowledge acquisition, known as epistemic beliefs. Understanding epistemic beliefs is crucial, as they shape the way individuals evaluate and interpret information, including alternative facts and conspiracy theories. This study was designed to address this paucity of research by examining the association between the use of classical psychedelics and the tendency to subscribe to alternative facts. We postulated that recent use of classical psychedelics would correlate with an increased inclination towards the belief in alternative facts, the conviction that facts are largely politically driven, and a tendency to rely on intuition rather than empirical evidence when forming beliefs. Alcohol was chosen as a reference substance given its well-documented pro-social and trust-enhancing effects^26,29^. Consequently, an inverse association was anticipated in this context.

The specific rationale for examining the recency of psychedelic use in relation to non-conformist beliefs was based on existing literature on the “afterglow” phenomena post-psychedelic experience, characterized by heightened emotional reactivity, divergent thinking and it’s time-varying nature^38^. We opted to focus on recency rather than the intensity or subjective experiences of the psychedelic event for several reasons. First, the afterglow effect's temporal dynamics provide a more concrete and measurable variable, allowing for more robust statistical analysis. Second, recency of use can be considered a more objective and quantifiable parameter compared to the inherently subjective nature of intensity, which may vary significantly among individuals. Lastly, by focusing on recency, we aimed to control for potential confounding variables that might be associated with the subjective experience's multifaceted nature.

## Method

### Participants

The participants were recruited through e-mail invitations sent out to a pool of people who had participated in a previous study examining the links between psychedelic use and schizotypy^39^. The recruitment for the present study was done before the COVID19 pandemic, over a period between January and May 2019. No compensation was given in direct relation to participating in the present survey.

The study employed a cross-sectional approach, targeting a demographic comprised primarily of healthy young adults in Sweden. We utilized G*Power v3 to calculate an appropriate sample size for detecting small-to-medium effect sizes. A sample size of at least 150 participants was deemed appropriate for the study.

A large variability in drug use patterns was observed with many people using drugs concomitantly: out of 392 participating subjects 376 people reported having used alcohol at least once, 324 used cannabis, 224 used MDMA, 97 used opiates, 233 used psychedelics, 212 used stimulants, and 341 reported uses of tobacco, and 16 reported not using any drugs.

The survey was administered in Swedish and English. All participants provided informed consent prior to participation. The study was approved by the Swedish Ethical Board (DNR: 2018/1040-31).

### Procedure

Recruitment occurred through web-based announcements on social media services, forums which were expected to include our target population (Facebook and Reddit groups discussing scientific and recreational use of psychedelic drugs, drug policy, substance-related issues and disorders), as well as general platforms designed to recruit research subjects, as a part of a larger study at Karolinska Institutet focusing on schizotypy. The data from several assessments (see Supplement) were merged in order to maximize the sample size. Potential sampling bias in relation to different platforms was addressed elsewhere with no apparent evidence for such being identified^39^. However, due to the specific focus on drug use, it is important to recognize that the total study sample may not be representative of the general population.

### Materials

In our endeavour to address the central research question, we employed two primary instruments: the Conspiracy Mentality Questionnaire and the Epistemic Belief Scale. We had a total of 392 participants, with a gender breakdown of 29% male, 41% female, and 30% who chose not to disclose their gender, complete the Conspiracy Mentality Questionnaire. The age range for this group was between 15 and 67 years, with a mean age of 28 years and a standard deviation of 7.2.

The Epistemic Belief Scale was completed by a sample of 305 participants. The gender distribution for this group was 23% male, 40% female, and 37% who did not provide their gender. The participants’ ages ranged from 18 to 67 years, with a mean age of 28 years and a standard deviation of 7.4.

In addition to these primary measures, the participants also filled out a series of trait questionnaires relating to psychopathology and personality, along with questions pertaining to their usage of drugs. Comprehensive demographic data, including age, gender, and psychiatric diagnoses, were collected as well. Data on validation of the Swedish versions of the instruments can be found in the Supplement.

Assessment of the factor structure in Swedish- and English-speaking groups revealed that the strict invariance assumption is met for the EBS, while the metric invariance assumption (with constrained loadings) is met for the CMQ (Supplement).

#### Primary outcomes

*The Conspiracy Mentality Questionnaire (CMQ)* is a measure of individual differences in conspiratorial thinking and ideation^40^, which for the purpose of this study was used to measure beliefs in alternative facts. CMQ consists of five statements (Table 1) that participants can rate their likelihood of agreeing with on a 11-point Likert-type scale, ranging from 0% (certainly not) to 100% (certain). For the main analysis, a mean score for all five items was calculated, while the exploratory analysis included the individual scores for each subitem. The subitems will be referred to as (1) misinformation, (2) politics, (3) monitoring, (4) hidden connections and (5) secret organizations.

**Table 1:** Conspiracy mentality questionnaire.

1. I think that many very important things happen in the world, which the public is never informed about
2. I think that politicians usually do not tell us the true motives for their decisions
3. I think that government agencies closely monitor all citizens
4. I think that events which superficially seem to lack a connection are often the result of secret activities
5. I think that there are secret organizations that greatly influence political decisions

The CMQ has previously been validated in England, Ireland, Germany and Turkey^40^. Examinations of the factorial structure supports that conspiracy mentality is a one-dimensional construct that is stable over time and has been shown to have convergent, discriminant, and predictive validity^40^.

The present study used a previously constructed, but unvalidated Swedish version of the CMQ (Supplement). For this reason, a psychometric evaluation was performed (Supplementary Materials). Construct reliability and validity could be confirmed for a modified model, where several of the observed variables were allowed to correlate.

*The Epistemic Belief Scale (EBS)* consists of twelve statements (Table 2) that assess how people treat knowledge and process information, an important foundation of beliefs^41^. The twelve statements are divided into groups of four, generating three facets of epistemic beliefs; Faith in Intuition for facts (FI-facts), Need for evidence and Truth is political. The two first facets (FI-facts, Need for evidence) relate to how much bearing an individual puts on feelings compared to evidence when forming opinions. The third facet (Truth is political) includes items regarding the conviction that facts are influenced by politics^41^. Participants rated their likelihood of agreeing with each statement by using a 5-point Likert-type scale ranging from disagree completely to agree completely. All the three EBS facets have shown acceptable scale reliability and test re-test reliability^41^.

**Table 2:** Epistemic belief scale.

Faith in intuition for facts	
Feel1	I trust my gut to tell me what’s true and what’s not
Feel2	I trust my initial feelings about the facts
Feel3	My initial impressions are almost always right
Feel4	I can usually feel when a claim is true or false even if I can’t explain how I know

Need for evidence	
Evid1	Evidence is more important than whether something feels true
Evid2	A hunch needs to be confirmed with data
Evid3	I trust the facts, not my instincts, to tell me what is true
Evid4	I need to be able to justify my beliefs with evidence

Truth is political	
Poli1	Facts are dictated by those in power
Poli2	What counts as truth is defined by power
Poli3	Scientific conclusions are shaped by politics
Poli4	“Facts” depend on their political context

Sufficient convergent and discriminant validity has been confirmed using structural equation modelling with several other measures, including conspiracist ideation, which was found to be positively associated with FI-facts and Truth is political, and negatively with Need for evidence^41^. For the present study, we translated the EBS scale to Swedish and performed a validation analysis confirming its construct validity and a good fit with the data.

#### Traits related to psychopathology

*Peters *et al*. Delusions Inventory (PDI)* was designed to measure delusional ideation in the normal population^42^. It is comprised of 21 items with three domains that measures different levels of Distress, Preoccupation and Conviction. Each item is a yes or no question, however, if someone answers yes, they are asked to mark their answer for the amount of Distress, degree of Preoccupation and the level of Conviction that a certain belief/experience may have, on a 5-point scale^42^. The scale has adequate internal consistency, concurrent validity and test–retest reliability. In the present study only yes or no alternatives were used (no subitem rating was performed).

*Oxford-Liverpool Inventory of Feelings and Experiences (O-LIFE)* consists of four subscales and 104 items in total. Each subscale is thought to measure different facets of the schizotypy construct in the normal population: Unusual Experiences (30 items) measures different types of cognitive and perceptual disturbances such as magical thinking and hallucinations. Cognitive Disorganization (24 items) relates to executive functioning, such as attention-deficits, disorganized speech as well as social anxiety. Introvertive Anhedonia (27 items) measures dissatisfaction or lack of interest in exciting, social and physical engagements. Impulsive Nonconformity (23 items) taps into odd behaviours that relate to both impulsivity and antisocial behaviour. All items are yes or no questions. All scales have been found to have adequate internal consistency and test–retest reliability^43^.

*The Adult ADHD Self-Report Scale (ASRS)* was used to evaluate (subclinical and clinical) symptoms of attention deficit hyperactivity disorder (ADHD) over the full range in the population. The ASRS consists of 18 items that are divided into two parts that are consistent with the Diagnostic and Statistical Manual for Mental Disorders (DSM-IV) criteria for ADHD in adults. Each question can be answered on a 5-point Likert-type scale that ranges from *never* to *very often* in frequency. Answers can be scored as either *positive* or *negative* and each question has a different threshold. Four or more answers coded as *positive* in Part A (items 1- 6) is indicative of occurrence of ADHD. The ASRS was developed by the World Health Organization (WHO)^44^. ASRS is normally distributed in the population^45^ and has been shown to have good internal reliability, test–retest reliability, as well as concurrent validity with a rater-administered ADHD-scale^46^.

*The Ritvo Autism and Asperger Diagnostic Scale-Revised (RAADS-R)* was used to appraise symptoms of autism spectrum disorder (ASD). RAADS-R is a 50-item scale that has been developed to diagnose ASD in adults. It is normally distributed in the population and has demonstrated excellent psychometric properties^47^.

#### Drug use

The participants were asked a number of questions related to the use of different drugs; alcohol, cannabis, MDMA, opiates (e.g. heroin, morphine, opium), psychedelics (e.g. LSD, magic mushrooms/psilocybin, ayahuasca/DMT), stimulants (e.g. amphetamine, ephedrine, cocaine) and tobacco. For each drug, the subjects were required to answer how many times they had used it (frequency) by selecting one of 5 scale points; never, 1–3 times, 4–10 times, 11–50 times or more than 50 times. Additionally, participants were asked when their last time of intake had been (recency) by selecting one of 8 scale points; never, less than 24 h ago, less than 3 days ago, less than a week ago, less than a month ago, less than a year, less than 5 years ago or more than 5 years ago. The analysis was specifically focused on the variables that represent a more nuanced understanding of exposure to better tease out effects in a manner that transcends lifetime use and is not confounded by age. However, the participants were also asked to submit at what age they used a drug for the first time.

#### Personality

*The short version of the Big Five Personality Inventory (BFI-S)* incudes 15 items and measures the five factor model constructs: openness, conscientiousness, extraversion, agreeableness and neuroticism. Participants rate how much they agree with each statement using a 7-point scale ranging from 1 (strongly disagree”) to 7 (strongly agree). Both convergent and discriminant validity with other personality measures has been demonstrated for the present scale^48^.

### Statistical analyses

Prior to statistical analysis, all data was screened for outliers with high influence, using Cook’s Distance. A Cook’s distance > 1.0 was considered large. No outliers were detected. Normality of the distributions was analysed for the total score of CMQ and the three facets of EBS: 1—Faith in Intuition for facts (FI-facts), 2—Need for evidence and 3—Truth is political). The Shapiro–Wilk test for normality indicated that the data for the total score of CMQ was not normally distributed (*p* < .001) although an overview of histogram and distribution of residuals revealed sufficient normality for the analysis employing linear modelling. Similarly, regarding the EBS facets, normal distribution of residuals was considered sufficient to support the planned analysis, and no additional transformations were carried out.

In order to investigate the relationship between recent psychedelic drug use, alcohol use and beliefs in alternative facts, as measured by the total score of CMQ, three linear regression models were fitted stepwise. The first model included recent use of psychedelics as a single-variable predictor of beliefs in alternative facts (the outcome variable total score on the CMQ). Next step included verification of significant findings by means of additional adjustments for demographics, psychiatric conditions, and concomitant drug use. In the last step, an exploratory analysis was performed investigating the relationship between frequent use of the different drugs of reference and epistemic beliefs.

To test the hypotheses regarding the effect of recent use of psychedelics on the three facets of epistemic beliefs (FI-facts, Need for evidence, Truth is political), as measured with EBS, several linear regression models were fitted stepwise, employing a similar strategy. The first step included three single-variable models with recent use of psychedelics, as predictors, as measured by the latest intake of the drug. Each model had a different EBS factor as an outcome variable. Next steps followed the same procedure as for the CMQ analysis.

To check for multi-collinearity, the variance inflation factors (VIFs) were inspected. A VIF < 1 was considered satisfactory.

To further investigate the associations between the use of psychedelics and beliefs in alternative facts, a comparison between two groups was also carried out. The group of psychedelic users (N = 233) included people who had reported having used psychedelics at least once in their lifetime. The second group consisted of subjects that reported not using psychedelics (non-users, N = 159). In order to establish if there were any significant differences between the groups in relation to the total score on CMQ and its subitems, independent samples t-tests were performed. The same procedure was performed for the three facets of the EBS (N_total_ = 305, N_users_ = 180, N_non-users_ = 125).

Following the main analyses, we also reported inherent associations among the variables under investigation within the sample of psychedelic users (n = 180), leveraging cross-correlation network plot.

Finally, two correlation matrices were estimated. The first included measures of recent use and the different subscales of BFI-S as well as the composite score delusion proneness, in order to investigate the associations between different drug use patterns and personality. The second matrix included raw-scores for psychopathology (O-LIFE, PDI, RAADS, ASRS) as well as the total score of CMQ and the three facets of EBS, in order to explore the associations between our main measures and psychopathology.

To correct for multiple comparisons, the false discovery rate procedure was employed, as represented in-text with the variable Pcorr.

The factor analyses were performed using Lavaan package version 0.6–5^49^ in R Studio version 3.6.2^50^. All other statistical analyses were carried out in Jamovi (Version 1.1.9.0) and replicated independently in R Studio version 3.6.2.

### Ethical consideration

The study was approved by the Swedish Ethical Board (Etikprövningsmyndigheten, DNR: 2018/1040-31) and fully adhered to the Declaration of Helsinki principles^51^. To our knowledge, there were no physical risks associated with participating in this study and steps were taken to ensure the participants’ psychological well-being. Prior to starting the survey, informed consent was given by all participants. This included information that described the purpose of the study, which was formulated as an investigation of the relationship between cognitive styles and the perception of one’s environment in relation to different kinds of drug use patterns.

## Results

### Drug use of psychedelics and beliefs in alternative facts measured by CMQ

Results indicated that recent use of psychedelics was significantly associated with beliefs in alternative facts as measured with CMQ (β_std_ = .174, t = 3.49, *p* < .001, *F*(1, 390) = 12.7, *R*^2^ = .0317, *R*^2^*Adjusted* = .0292) in a single-predictor model. The effect remained significant even when repeating the analysis only in the group of psychedelic users (N = 233, β_std_ = .147, t = 2.26, *p* = .024, *F*(1, 231) = 5.135, *R*^2^ = .0217, *R*^2^*Adjusted* = .0175).

The second model included control variables for age, sex and psychiatric conditions. Results indicated that recent use of psychedelics was still a significant predictor of beliefs in alternative facts in the whole sample (N = 392, β_std_ = .212, t = 3.34, *p* = .001, *F*(4, 268) = 3.2, *R*^2^ = .046, *R*^2^*Adjusted* = 0.031) and in a subsample of psychedelic users (*β*_*std*_ = .195, t = 2.316, *p* = .022, *F*(4, 134) = 3.425, *R*^2^ = .093, *R*^2^*Adjusted* = .0657).

**Figure 1:**
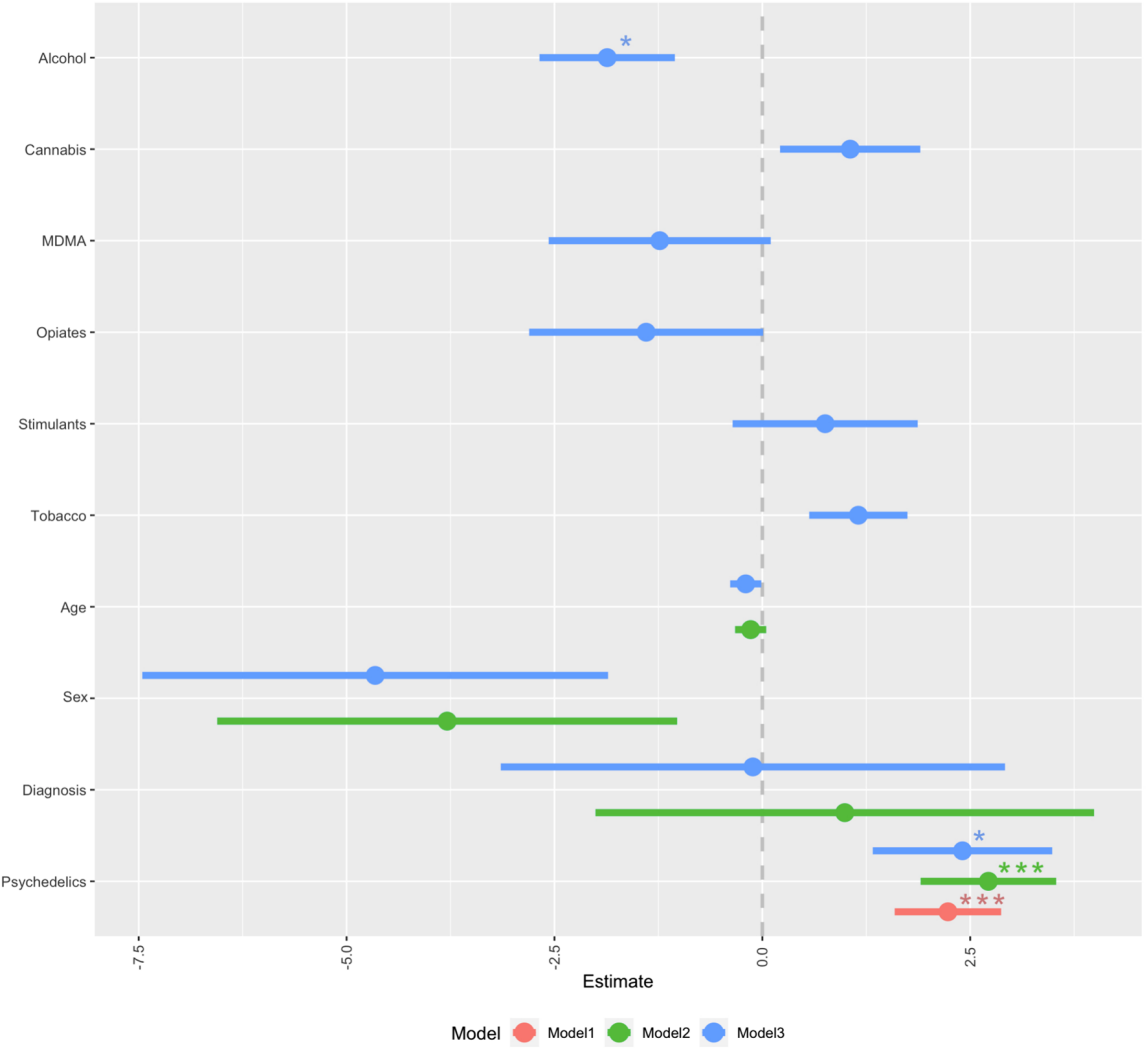
Coefficient plot of multiple regression models for the predictors of mean score on the CMQ. Estimate: non-standardized coefficients. Model1: minimal (psychedelic use and intercept), Model2: demographic adjustment, Model3: full model adjusted for concomitant drug use. Error bars denote 95% confidence intervals.

In a third model, controlling for demographics and concomitant drug use, recent use of psychedelics (β_std_ = .187, t = 2.31, *p* = .027) and alcohol (*β*_*std*_ =− .142, t = − 2.93, *p* = .023), were differentially associated with beliefs in *alternative facts* (*F*(10, 262) = 2.425, *R*^2^ = .08, *R*^2^*Adjusted* = .05) in the whole sample. However, these effects were only marginally significant when limiting the analysis by those who explicitly reported use of psychedelics (Psychedelics: β_std_ = .155, t = 1.66, *p* = .0986; Alcohol: *β*_*std*_ = 0.155, t = 1.91, *p* = .0587;* F*(10, 128) = 2.075, *R*^2^ = 1.39, *R*^2^*Adjusted* = .07). The models can be viewed in Fig. 1.

Of note, the discovered associations were still present (although became marginally significant) even when introducing PDI into the final model (Psychedelics: *β*_*std*_ = .154, *p* = .05, Alcohol: β_std_ = − .12, *p* = .056).

A separate model included beliefs in *alternative facts* as the dependent variable and all drugs of reference, as measured by frequent use of the drug, and control variables was fitted. The individual predictor *stimulants* (*β*_*std*_ = .2215, *p* = .028) was the only significant predictor of beliefs in *alternative facts* (*F*(10, 262) = 2.57, *p* = .006, *R*^2^ = .0893, *R*^2^*Adjusted* = .0545, see Supplement).

Limiting the analyses with a sample of subjects without psychiatric diagnosis (n = 200, 73.26%) yielded similar results for the effects of psychedelic use (Model1 [psychedelic use and intercept]: *β*_*std*_ = .172, *p* = .02, Model2 [demographic adjustment]: β = .224, *p* = .002, Model3 [full adjustment for concomitant drug use, marginally significant]: *β*_*std*_ = .15, *p* = .1) and alcohol (Model3 [full adjustment for concomitant drug use, marginally significant]: *β*_*std*_ =− .1625, *p* = .023).

A similar pattern of associations was observed when Swedish and Non-Swedish groups were analysed separately. Of note, Swedish sample scored significantly lower on CMQ compared to the non-Swedish sample (47.18 ± 20.98 and 53.08 ± 22, respectively; t_382_ = 2.71, *p* = .007). See Supplement for details.

Adjustment of the models for mother tongue did not result in any substantial deviations in the reported results.

### Difference between psychedelic drug users and non-users (group comparisons): CMQ

**Figure 2:**
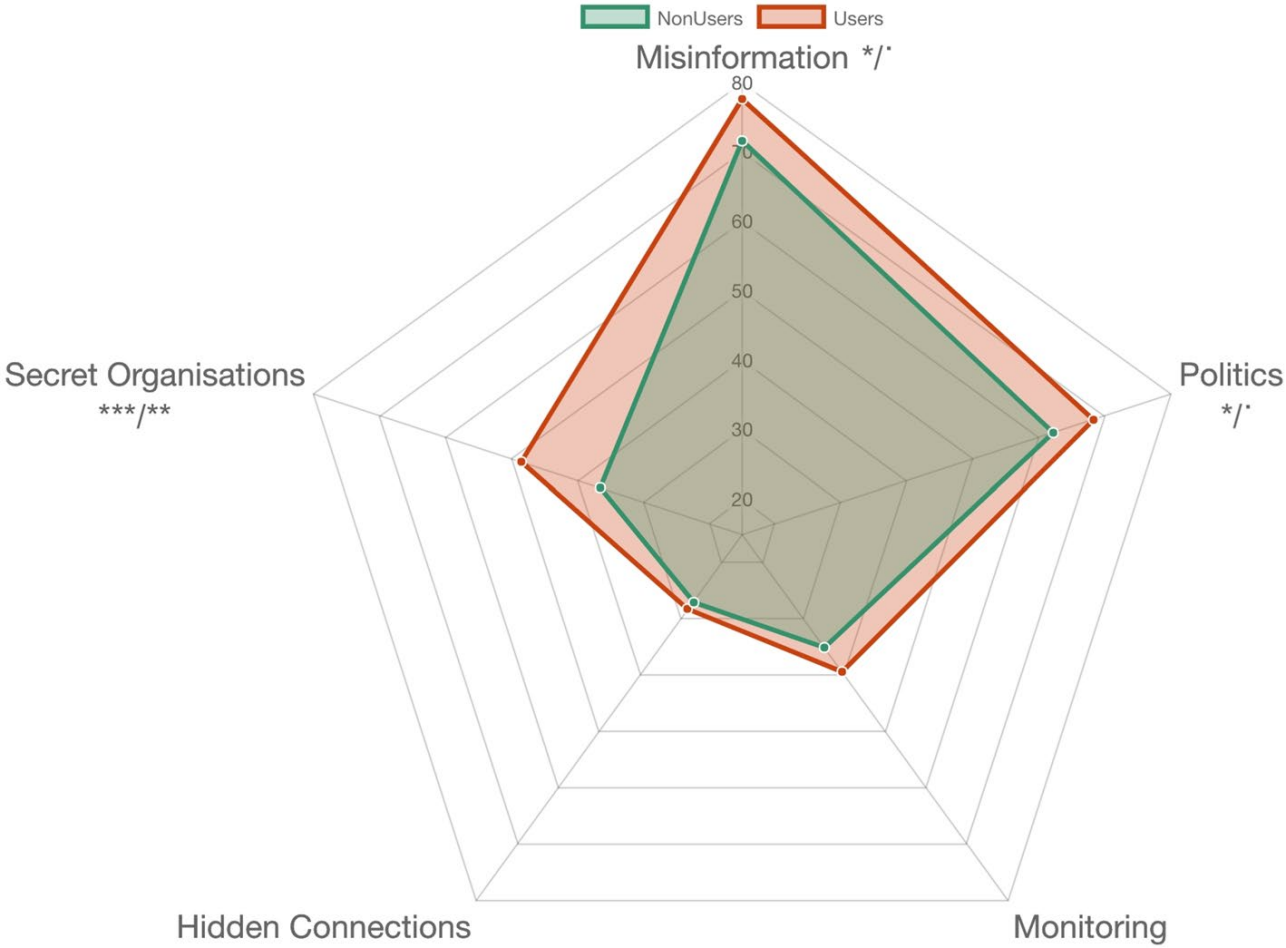
Differences in CMQ mean scores between psychedelic drug users and Non-Users. p/p_corr_—*p*-value, unadjusted and corrected for multiple testing: * < .05; ** < .01; *** < .001.

The comparison showed that psychedelic users (M = 52.42, SD = 21) scored significantly higher than non-users (M = 46.44, SD = 22) on the CMQ (t(329.63) = 2.685, *p* = .008, Pcorr = .0076). The effect size, however, was relatively small, d(95% CI) = .28 (0.075–0.48). The comparison between the different subitems of the CMQ showed significant differences (Fig. 2). Psychedelic users scored higher than non-users on the first subitem, Misinformation (t(290) = 2.27, *p* = .023, d = .235(0.032–0.44)), second subitem, Politics (t(336.91) = 2.25, *p* = 0.025, d = 2.23(0.029–0.43)), as well as on the fifth subitem, secret organizations (psychedelic users: M = 48.45, SD = 31.4; non-users: M = 36.48, SD = 32.45; t(346.73) = 3.66, *p* < .001, d = .37. After adjusting the results for multiple comparisons, only the last difference remained significant (Secret Organisations, pcorr = .001) and the other two items showed difference of marginal significance (Misinformation, pcorr = .09; Politics, pcorr = .09).

### Use of psychedelics and Epistemic beliefs

#### FI-facts

The first single-variable regression model did not confirm any significant relationship between recent use of psychedelics (*β*_*std*_ = .0222, *p* = .699) and the EBS factor *FI-facts* (*F*(1, 303) = .149, *p* = .699, *R*^2^ = .0005, *R*^2^Adjusted = .00281). There were no significant findings in the exploratory analysis.

#### Need for evidence

The first single-variable regression model did not confirm any significant relationship between recent use of psychedelics (*β*_*std*_ = − .0897, *p* = .118) and *Need for evidence* (*F*(1, 303) = 1.964, *p* = .1621, *R*^2^ = .00644, *R*^*2*^*Adjusted* = .003161). The exploratory analysis, showed that frequent use of psychedelics (*β*_*std*_ = − .130, *p* = .024) significantly predicted the outcome variable Need for evidence in a single-predictor model (*F*(1, 303) = 4.7, *p* = .03094, *R*^2^ = .01527, *R*^*2*^*Adjusted* = .01202). After controlling for demographics, frequent use of psychedelics (*β*_*std*_ = − .2708, *p* < .001) remained significant together with the control variable male sex (*β*_*std*_ = − .4795, *p* = .002). Together they significantly predicted a low score on the outcome variable (*F*(4, 187) = 4.78, *p* < .001, *R*^2^ = .0927, *R*^2^*Adjusted* = .0733). However, after introducing adjustments for concomitant drug use, only male sex (*β*_*std*_ = − .04766, *p* = .003) remained a significant predictor (*F*(10, 181) = 2.43, *p* = .010, *R*^2^ = .118, *R*^2^*Adjusted* = .0695).

#### Truth is political

**Figure 3:**
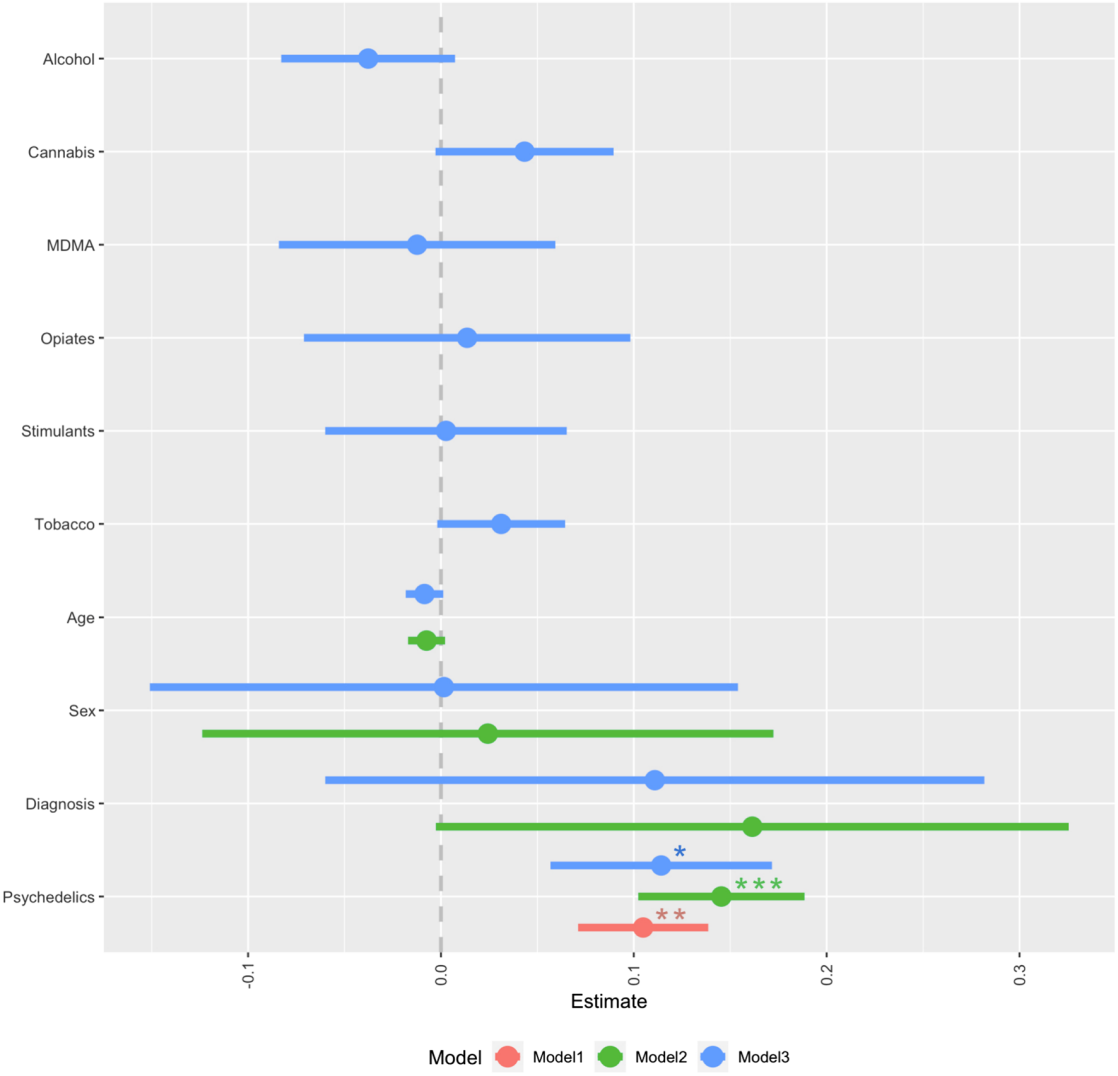
Coefficient plot of multiple regression models for the predictors of the EBS score: Truth Is Political. Model 1: minimal (psychedelic use and intercept), Model 2: demographic adjustment, Model 3: full model adjusted for concomitant drug use. Error bars denote 95% confidence intervals and *—*p* < .05; **—*p* < .01; ***—*p* < .001.

The first single-variable model showed that recent use of psychedelics (*β*_*std*_ = 0.176, *p* = .002) significantly predicted a high score on the outcome variable Truth is political (F(1, 303) = 9.64, *p* < .001, R^2^ = .031, R^2^Adjusted = .028). Recent use of psychedelics remained a significant predictor after controlling for demographics in a second model (*β*_*std*_ = .251, *p* < .001; F(4, 187) = 3.925, *p* = .004, R^2^ = .078, R^2^Adjusted = .058), as well as in a third model that controlled for concomitant drug use (psychedelics: *β*_*std*_ = .197, *p* = .048; F(10, 181) = 1.86, *p* = .05, R^2^ = .093, R^2^_Adjusted_ = .043). All three models can be viewed in Fig. 3.

Of note, introducing PDI into the last model did not change the result, however, the investigated effect of psychedelic use on the facet “truth is political” became only marginally significant (*β*_*std*_ = 164, *p* = .095).

In the analyses of a subsample of those without psychiatric conditions (n = 143, 74.48%), the result was similar, except for the last model (Truth is political|Model1: *β*_*std*_ = .2, *p* = .017, Model2: *β*_*std*_ = .227, *p* = .008, Model3: *β*_*std*_ = 0.05, *p* = .647).

A similar pattern of associations was observed when Swedish and Non-Swedish groups were analysed separately. See Supplement for details.

### Difference between psychedelic drug users and non-users (group comparisons): EBS

**Figure 4:**
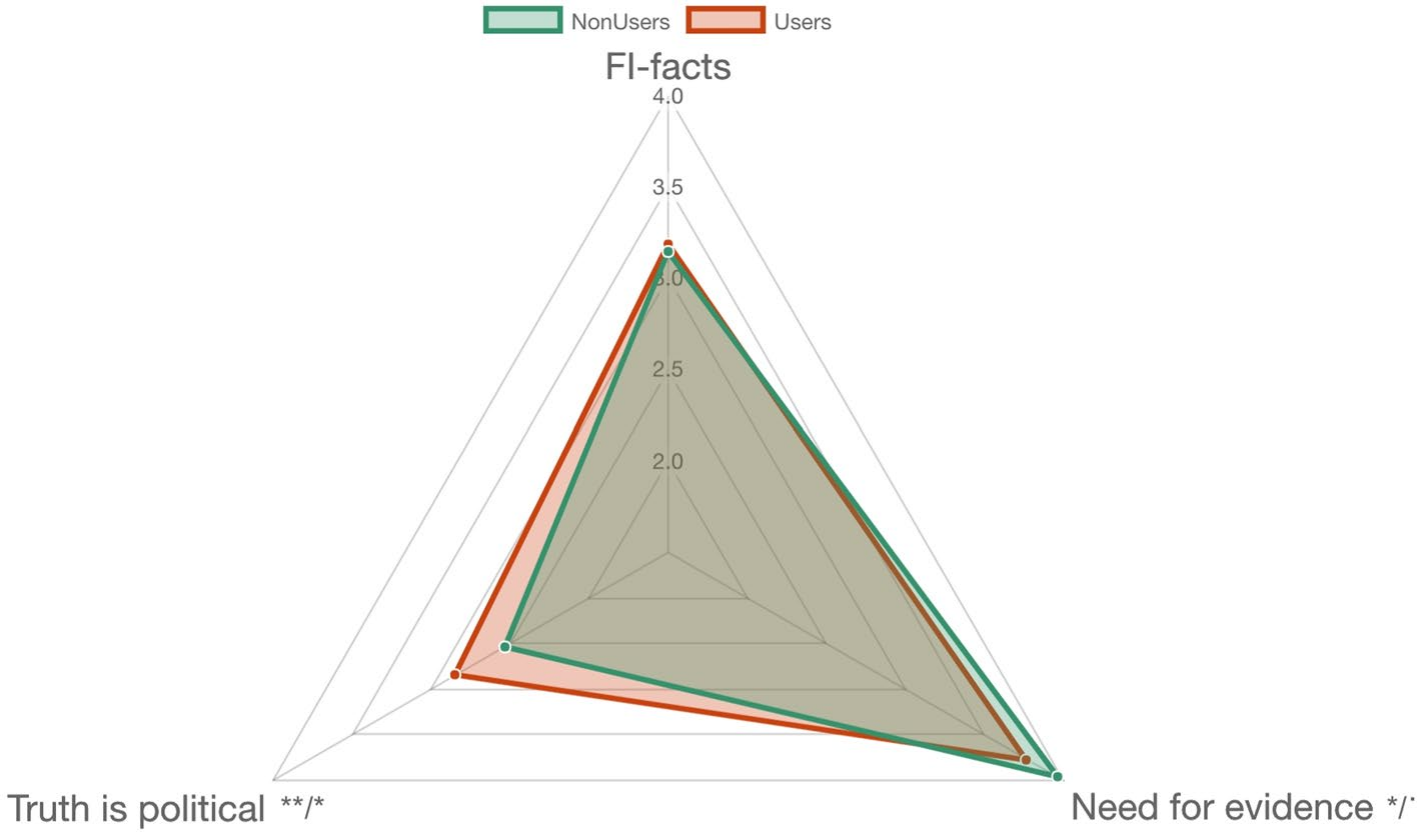
Differences in mean EBS scores between psychedelic drug users and non-users. FI-facts—Faith in intuition for facts. p/p_corr_—*p*-value, unadjusted and corrected for multiple testing: * < .05; ** < .01.

In order to further explore the differences between *psychedelic users* (*N* = 180) and *non-users* (*N* = 125), the same approach as the one used for the CMQ subitems was employed, for the different facets of EBS. As can be seen in Fig. 4, the results showed that there was a difference in the score for EBS factor *Need for evidence*, where *psychedelic users* scored lower (non-transformed *M* = 3.77, *SD* = 0.859) than *non-users* (non-transformed *M* = 3.96, *SD* = 0.72). This difference, however, was only marginally significant when adjusting the result for multiple testing (*t*(292.67) = 2.08, *p* = .0376, *p*_*corr*_ = .075).

The difference on the factor *Truth is political*, on the other hand, was consistently significant (*t*(270.66) = 2.575, *p* = .006, *p*_*corr*_ = .0177)), on which *psychedelic users* scored higher (*M* = 2.85, *SD* = 1.014) than *non-users* (*M* = 2.53, *SD* = 0.992).

### Exploratory cross-correlation network analysis

**Figure 5:**
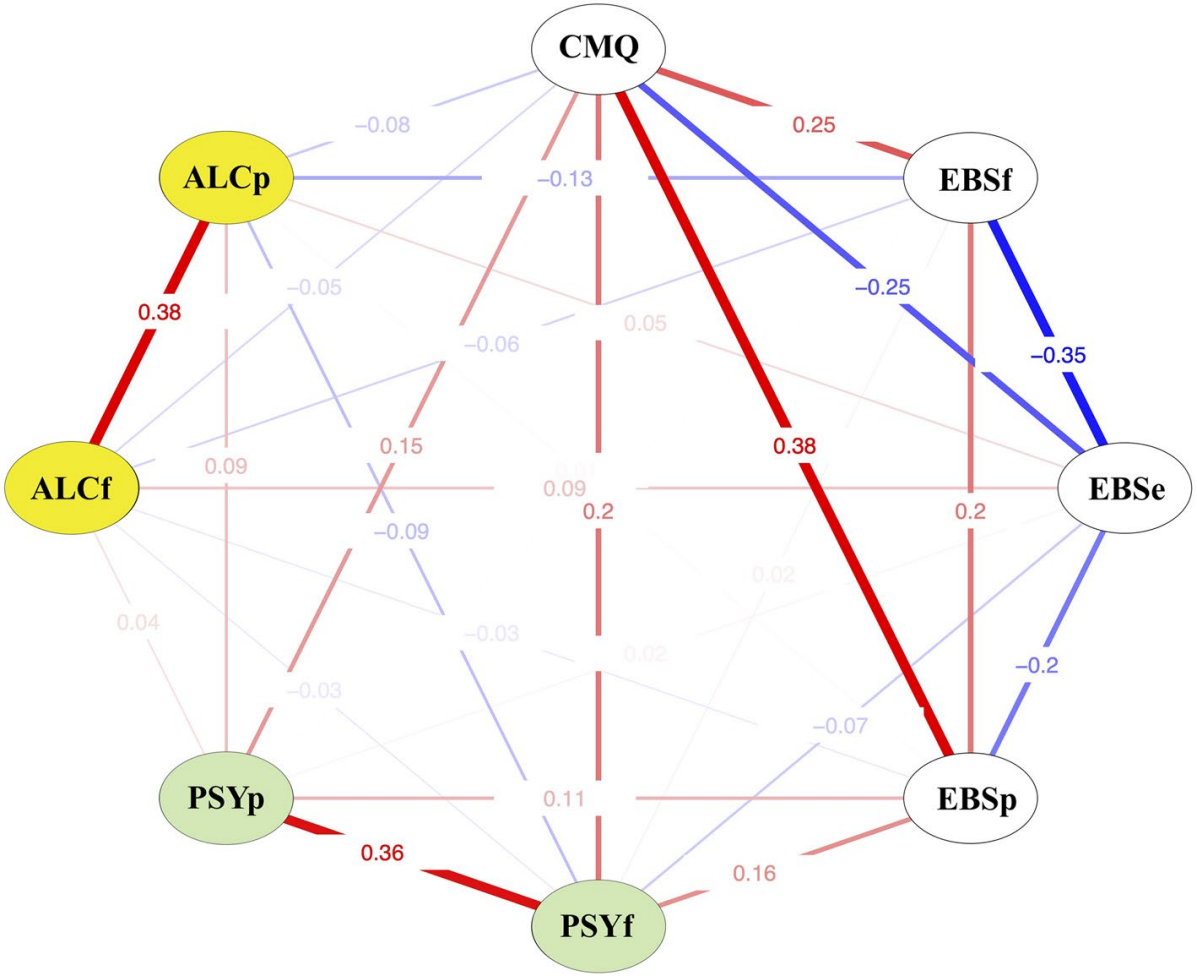
Cross-correlation network for the main investigated variables in a sample of psychedelic users (n = 180). Edges with numerical values represent Pearson correlation coefficients. The abbreviations used are as follows: CMQ—Conspiracy Mentality Questionnaire, EBS_f_ – *Faith in intuition for facts,* EBS_e_—*Need for evidence,* EBS_p_—*Truth is political*; ALCp/f—temporal proximity and frequency of alcohol use, PSYp/f—temporal proximity and frequency of classical psychedelic use.

To further scrutinize the inherent associations among the variables under investigation within our psychedelic user sample (n = 180), we conducted a cross-correlation matrix analysis leveraging the Pearson r coefficient. The resultant patterns were found to be congruent with those deduced from our regression analyses and group comparisons (Fig. 5).

### Personality and drug use

In order to investigate the relationships between drug use and personality, two correlation matrices containing the measures of recent and frequent use of all drugs of reference were created, both containing variables for the subscales of BFI-S (*openness, conscientiousness, extraversion, agreeableness* and *neuroticism*). We have also explored corresponding associations of the investigated variables with the psychopathology-related traits *delusion proneness and schizotypy*, as measured by PDI and O-LIFE, respectively. Significant findings will be reported here, the rest can be found in the Supplement.

#### Openness

Both recent and frequent use of psychedelics (recent: *r*(392) = .248, *p* = .005); frequent: *r*(392) = .255, *p* < .001), MDMA (recent: *r*(392) = .178, *p* < .001); frequent: *r*(392) = .222, *p* < .001) and tobacco (recent: *r*(392) = .173, *p* < .001); frequent: *r*(392) = .157, *p* = .002) was found to be significantly correlated with *openness*. Additionally, frequent use of cannabis (*r*(392) = .221, *p* < .001) and recent use of stimulants (*r*(392) = .230, *p* < .001) significantly correlated with *openness*.

#### Conscientiousness

Significant negative relationships were found for *conscientiousness*, with recent and frequent use of MDMA (recent: *r*(392) = − .112, *p* = .027); frequent: *r*(392) = − .107, *p* = .034), recent use of stimulants (*r*(392) = − .120, *p* = .018) as well as frequent use of cannabis (*r*(392)  = − .119, *p* = .019) and psychedelics (*r*(392) = − .139, *p* = .006).

#### Extraversion

Recent use of alcohol (*r*(392) = .124, *p* = .014) was the only variable showing a significant relationship with *extraversion*.

#### Agreeableness

*Agreeableness* was found to be significantly associated with recent use of stimulants (*r*(392) = .116, *p* = .021).

#### Neuroticism

Both recent and frequent use of psychedelics (recent: *r*(392) = − .130, *p* = .010); frequent: *r*(392) = − .171, *p* < .001), MDMA (recent: *r*(392) = − .110, *p* = .030); frequent: *r*(392) = − .159, *p* = .002) and tobacco (recent: *r*(392) = − .100, *p* = .049); frequent: *r*(392) = − .116, *p* = .022) was found to have significant negative associations with *neuroticism*.

####  Delusion-proneness

Both recent and frequent use of psychedelics was found to be significantly associated with *delusion proneness* (recent: *r*(273) = .169, *p* = .005); frequent: *r*(273) = .124, *p* = .040). Additional significant relationships was found for recent use of MDMA (*r*(273) = .134, *p* = .027) and frequent use of cannabis (*r*(273) = .124, *p* = .040).

### Traits related to psychopathology

In order to investigate the relationship between our primary measures (CMQ, EBS) and psychopathology-related traits, a second correlation matrix was created (Table 3). This included the total score for CMQ, the three facets of EBS (*FI-facts, Need for evidence, Truth is political),* the total score of ASRS, RAADS, total score of PDI, and the different subscales of O-LIFE (*Unusual Experiences, Cognitive Disorganization, Introvertive Anhedonia, Impulsive Nonconformity*).

**Table 3:** Correlation matrix for scores on the CMQ, facets of EBS, PDI, subscales of O-LIFE, ASRS and RAADS.

	PDI	OLIFE UE	OLIFE CD	OLIFE IA	OLIFE IN	ASRS	RAADS
CMQ	Pearson’s r. 398***	.286***	.128	.181*	.276***	.108	.040
EBS_f_	Pearson’s r. 347***	.295***	.106	.045	.149*	.030	− .024
EBS_e_	Pearson’s r-. 282***	− .266***	− .116	.053	− .089	− .071	− .035
EBS_p_	Pearson’s r. 300***	.227**	.154*	.173*	.297***	.155*	.030

*N* = 192. * = *p* < .05, **p < .01, ***p < .001. EBS_f_—Faith in intuition for facts, EBS_e_—Need for evidence, EBS_p_—Truth is political.

## Discussion

The primary aim of the present study was to investigate the relationship between patterns of drug use and users' beliefs, with a particular emphasis on classical psychedelics. The focus was to examine potential associations between psychedelic exposure and the inclination to affirm alternative views related to three distinct epistemic beliefs, as measured by the Epistemic Belief System (EBS): (1) reliance on intuition for fact formation (Faith in intuition for facts), (2) tendency to corroborate assertions with empirical data (Need for evidence), and (3) perception of factual information as politically driven (Truth is political). In a similar way, we wanted to elucidate their relation to non-conventual beliefs (using CMQ). To further understand the complex associations between drug use patterns and beliefs, we also wanted to look at these relationships in connection to different personality and psychopathology-related traits.

The obtained results aligned with our expectations, showing a statistically significant association between recent and frequent psychedelic use and affirmations of alternative views, as assessed by the Conspiracy Mentality Questionnaire^40^. Correspondingly, recent alcohol consumption exhibited an inverse relationship with the endorsement of alternative views. Also noteworthy is the significant positive association between frequent stimulant use and alternative views. The yielded results retained significance after controlling for demographic factors and concomitant drug use, indicating that certain drug use patterns might be associated with accepting non-conventional beliefs, such as conspiracy theories, which previous research has tied to intuition-based rather than evidence-based beliefs^41^.

Contrary to our hypothesis, the study did not establish any association between psychedelic use and a preference for intuition over facts. Collectively, these findings suggest that psychedelic use may be linked to cognitive styles that question mainstream information sources rather than to outlandish conspiracy ideation^52^.

Previous literature has found connections between the endorsement of *alternative facts* and certain psychopathology-related traits, including facets like paranoia and schizotypy^53,54^. This finding has been confirmed in the present study as well showing significant associations between beliefs in alternative facts and facets of schizotypy. Of note, our group previously explored the link between psychedelic use and schizotypy and found that this association is largely explained by use of other drugs like stimulants^39^. However, the association with unconventional beliefs remained significant even after adjusting for other drug use, suggesting a specific link to psychedelic use.

Some^26,29^, but not all^55,56^ studies identified associations between alcohol use and pro-social behaviours and beliefs. In this context, our results showing negative association between alcohol use and beliefs in alternative facts provide certain support for this link. However, they must be interpreted with caution as only a minority of the participants reported to never have used alcohol. Of note, we also confirm previous findings demonstrating that alcohol use is associated with *extraversion*^7,9^.

Furthermore, the use of all investigated drugs except opiates and alcohol, revealed significant associations with high scores on the personality trait *openness*. The use of MDMA and stimulants showed negative associations with the trait *conscientiousness*. These results are in line with previous literature^8,9,57^. Scoring high on *openness* and low on *conscientiousness* is not merely associated with drug use but may also reflect *openness* toward unconventional views and non-conformity^33,34^.

It is again important to acknowledge that most (73.26%) of our subjects were healthy, normally functioning adults and that higher scores on the CMQ do not equal pathological *conspiracy ideation*. In fact, it could be hypothesized that the challenging of social and ideological norms can be triggered by real societal phenomena such as the revealing of widely spread surveillance programs, e.g. the leak of the NSA files^58^, or the lack of adequate legal frameworks protecting personal integrity and privacy on the internet^59^. In this perspective, a theoretical separation between the pathological grounded conspiracist beliefs and the unconventional healthy sceptic must be assumed. This may indicate that the results, in fact, rather reflect an existing spectrum of *non-conformist mentality*, which to a degree is dependent on contextual factors. This view is supported in our exploration of between-group differences in main facets of the CMQ. In line with this line of thought, *psychedelic users* scored significantly higher than *non-users* on two items (*secret organizations*, *misinformation*), however, the item *hidden connections* (“I think that events which superficially seem to lack a connection are often the result of secret activities.”) was not significant between the groups. Of note, it can be speculated that this particular item reflects an integral aspect of the pathological conspiracy construct, namely, the tendency to see patterns of extraordinary phenomena in everything^60^ or pareidolia^61^. The absence of between- group differences in this domain support the idea that the results may reflect differences in non-conformity and general scepticism toward official sources of information, rather than a conspiracy ideation per se.

The presence of a difference between users and non-users of psychedelics in relation to beliefs in alternative facts suggest a least two possible explanations: (a) individuals inclined towards psychedelic use might inherently affirm alternative views more than others, irrespective of the drug's effects, due to shared traits or environment, or (b) these individuals might develop such beliefs following psychedelic experiences that enhance their openness to experience and novel, unconventional ideas^35–37^. Further experimental investigations assessing non-conformist ideation and epistemic beliefs before and after the administration of psychedelics could provide further clarity on these drugs’ potential to significantly alter how people process information.

Taken together, our findings should not be misconstrued as advocating restrictive drug policies. Conspiracy ideation is a complex societal issue, more nuanced than a direct consequence of drug use, and could even be fuelled by societal alienation and the ostracization of users of certain drugs^62^. Hence, the stigmatization of psychedelic drug users and the resultant social polarization may cultivate the conditions that foster conspiracy ideation. Moreover, the increasing prevalence of psychedelic culture and scepticism towards mainstream media might be components of the same generational shift in attitudes, as some scholars suggest^63^.

It is important to acknowledge that there are several limitations that restrict the generalizability of our findings. First, self-reporting is an inevitable limitation when assessing unobservable behaviours like beliefs and attitudes. All scales measuring such constructs possess inherent limitations in terms of accuracy and reliability that are often beyond the reach of current scientific methodologies^64^. Specifically, reporting bias in relation to drug use have previously been found^65^, highlighting the need for objective measures. Unfortunately, for this study, such measures were unfeasible, making self-reported measures the only accessible tool. As such, the presence of bias in this study is plausible, wherein participants could either underreport or exaggerate their drug use habits.

Moreover, the study’s scope was primarily focused on the Swedish population, specifically within groups where recreational drug use was expected. This specificity inevitably limits the generalizability of our findings. Cultural, social, and political contexts play a significant role in shaping both drug use patterns and belief systems. Thus, the associations observed in this study, rooted in a particular socio-cultural context, may not hold true across different populations or cultures. There may be distinct patterns in drug use and alternative beliefs amongst diverse global communities, influenced by numerous variables such as societal norms, drug policies, and cultural attitudes towards unconventional beliefs. Consequently, our findings should be cautiously extrapolated beyond the studied demographics notwithstanding the confirmed invariance for the main study scales in Swedish and non-Swedish groups and equivalent main finding’s effect-sizes. Future studies with more diverse and representative samples are necessary to verify and extend our results.

The COVID-19 pandemic is another important contextual factor. It should be noted that data for this study were collected before any general restrictions were implemented in Sweden. This may be seen as a strength, as subsequent restrictions and social distancing measures may have affected responses. It also suggests caution for future replication attempts and indicates a potentially interesting area of future research exploring whether there are subpopulations particularly sensitive to such events.

This study tried to tease out potential links between drug use and beliefs, which is why both measures of frequency and recency of use were collected. Including a measurement of quantity could have improved the validity of our measures. This would have refined our measure of exposure to the different drugs to a greater extent. However, estimating an average quantity of use over time may be problematic considering it being a heterogeneous and context-specific behaviour. Intra-individual differences regarding quantity might therefore be larger than the inter-individual differences, making it a poor measure in this case. We did include a measure regarding age and drug use. This was meant to complement the other measures with the purpose of evaluating differences between longer and heavier use, compared to shorter and more recreational forms of use. Unfortunately, this was not possible as the sample was quite homogenous. Most participants reported to have started using drugs at the same age, with a few “late starters” and no “early starters”. Therefore, the present study was focused on variables that represent a more nuanced understanding of exposure in order to better tease out its effects in a manner that is not confounded by age.

In conclusion, our results suggest that psychedelic use is associated with non-conformist views, specifically, with beliefs in *alternative facts* and the belief that facts are influenced by politics. To the best of our knowledge, these findings are unprecedented in connection to current scientific research. As a result, this study represents a first step towards filling the knowledge gap regarding the associations between different drug use patterns and information processing. Many unanswered questions remain, and a continuation of this research in a multidisciplinary direction would be beneficial in order to better understand the underlying neural and behavioural mechanisms related to these constructs. Do certain drugs exhibit lasting effects on people’s perception, beliefs and behaviour or do the discovered associations rather represent effects of non-conformist subpopulations that are more likely to question conventional socio-political norms, including use of illicit drugs? What is the role of alienation of illicit drug users on their non-conformist points of view? Answering these questions could provide valuable insights into factors surrounding drug abuse and inform evidence-based drug policy-making.

### Supplementary Information

Supplementary Information.

## Data Availability

The study and its hypotheses were pre-registered in the Open Science Foundation Framework database (https://osf.io/53jm2). The data and a script documenting steps to generate the plots are available on the first author’s GitHub page (https://github.com/alex-lebedev), “DAF” repository.

## References

[1] Bogenschutz MP (2015). Psilocybin-assisted treatment for alcohol dependence: A proof-of-concept study. J. Psychopharmacol..

[2] Carhart-Harris RL (2016). Psilocybin with psychological support for treatment-resistant depression: An open-label feasibility study. Lancet Psychiatry.

[3] Garcia-Romeu A, Griffiths R, Johnson M (2015). Psilocybin-occasioned mystical experiences in the treatment of tobacco addiction. Curr. Drug Abuse Rev..

[4] Gasser P, Kirchner K, Passie T (2015). LSD-assisted psychotherapy for anxiety associated with a life-threatening disease: A qualitative study of acute and sustained subjective effects. J. Psychopharmacol..

[5] Goodwin GM (2022). Single-dose psilocybin for a treatment-resistant episode of major depression. N. Engl. J. Med..

[6] Mitchell JM (2021). MDMA-assisted therapy for severe PTSD: A randomized, double-blind, placebo-controlled phase 3 study. Nat. Med..

[7] Fairbairn CE (2015). Extraversion and the rewarding effects of alcohol in a social context. J. Abnorm. Psychol..

[8] Fehrman E (2019). Personality Traits and Drug Consumption: A Story Told by Data.

[9] Flory K, Lynam D, Milich R, Leukefeld C, Clayton R (2002). The relations among personality, symptoms of alcohol and marijuana abuse, and symptoms of comorbid psychopathology: Results from a community sample. Exp. Clin. Psychopharmacol..

[10] Timmermann, C. *et al. Psychedelics alter metaphysical beliefs*. https://osf.io/f6sjk (2021) 10.31234/osf.io/f6sjk. 10.1038/s41598-021-01209-2 PMC8611059 34815421

[11] Griffiths RR, Richards WA, McCann U, Jesse R (2006). Psilocybin can occasion mystical-type experiences having substantial and sustained personal meaning and spiritual significance. Psychopharmacol. Berl..

[12] Kettner H, Gandy S, Haijen ECHM, Carhart-Harris RL (2019). From egoism to ecoism: Psychedelics increase nature relatedness in a state-mediated and context-dependent manner. Int. J. Environ. Res. Public. Health.

[13] Lyons T, Carhart-Harris RL (2018). Increased nature relatedness and decreased authoritarian political views after psilocybin for treatment-resistant depression. J. Psychopharmacol..

[14] Simonsson, O. *et al.* Effects of psychedelics on authoritarian attitudes revisited (under review). (2021). 10.1177/02698811261443677 PMC13310294 42068187

[15] Nichols DE (2016). Psychedelics. Pharmacol. Rev..

[16] Hari J (2019). Chasing the Scream: The Search for the Truth About Addiction.

[17] Blume S (2006). Anti-vaccination movements and their interpretations. Soc. Sci. Med..

[18] Douglas KM, Sutton RM (2018). Why conspiracy theories matter: A social psychological analysis. Eur. Rev. Soc. Psychol..

[19] Jolley D, Douglas KM (2014). The Effects of anti-vaccine conspiracy theories on vaccination intentions. PLoS ONE.

[20] Kata A (2010). A postmodern Pandora’s box: Anti-vaccination misinformation on the Internet. Vaccine.

[21] Kofta M, Soral W, Bilewicz M (2020). What breeds conspiracy antisemitism? The role of political uncontrollability and uncertainty in the belief in Jewish conspiracy. J. Pers. Soc. Psychol..

[22] van Prooijen J-W, Krouwel APM, Pollet TV (2015). Political extremism predicts belief in conspiracy theories. Soc. Psychol. Personal. Sci..

[23] Leiser D, Duani N, Wagner-Egger P (2017). The conspiratorial style in lay economic thinking. PLoS ONE.

[24] Sapountzis A, Condor S (2013). Conspiracy accounts as intergroup theories: challenging dominant understandings of social power and political legitimacy: Conspiracy accounts. Polit. Psychol..

[25] Bale JM (2007). Political paranoia v. political realism: On distinguishing between bogus conspiracy theories and genuine conspiratorial politics. Patt. Prejudice.

[26] Seid AK (2016). Social interactions, trust and risky alcohol consumption. Health Econ. Rev..

[27] Zmerli S, Newton K (2008). Social Trust and attitudes toward democracy. Public Opin. Q..

[28] Newton K (2001). Trust, social capital, civil society, and democracy. Int. Polit. Sci. Rev..

[29] Demant J, Järvinen M (2011). Social capital as norms and resources: Focus groups discussing alcohol. Addict. Res. Theory.

[30] Murphy A (2014). Using multi-level data to estimate the effect of social capital on hazardous alcohol consumption in the former Soviet Union. Eur. J. Public Health.

[31] Goldberg SB (2020). Post-acute psychological effects of classical serotonergic psychedelics: A systematic review and meta-analysis. Psychol. Med..

[32] Nour MM, Evans L, Carhart-Harris RL (2017). Psychedelics, personality and political perspectives. J Psychoact. Drugs.

[33] Carney DR, Jost JT, Gosling SD, Potter J (2008). The secret lives of liberals and conservatives: Personality profiles, interaction styles, and the things they leave behind. Polit. Psychol..

[34] McCrae RR (1996). Social consequences of experiential openness. Psychol. Bull..

[35] Carhart-Harris RL (2016). The paradoxical psychological effects of lysergic acid diethylamide (LSD). Psychol. Med..

[36] Lebedev AV (2016). LSD-induced entropic brain activity predicts subsequent personality change. Hum. Brain Mapp..

[37] MacLean KA, Johnson MW, Griffiths RR (2011). Mystical experiences occasioned by the hallucinogen psilocybin lead to increases in the personality domain of openness. J. Psychopharmacol..

[38] Evens R, Schmidt ME, Majić T, Schmidt TT (2023). The psychedelic afterglow phenomenon: A systematic review of subacute effects of classic serotonergic psychedelics. Ther. Adv. Psychopharmacol..

[39] Lebedev AV (2021). Psychedelic drug use and schizotypy in young adults. Sci. Rep..

[40] Bruder M, Haffke P, Neave N, Nouripanah N, Imhoff R (2013). Measuring individual differences in generic beliefs in conspiracy theories across cultures: Conspiracy mentality questionnaire. Front. Psychol..

[41] Garrett RK, Weeks BE (2017). Epistemic beliefs’ role in promoting misperceptions and conspiracist ideation. PLoS ONE.

[42] Peters E, Joseph S, Day S, Garety P (2004). Measuring delusional ideation: The 21-item Peters et al. delusions inventory (PDI). Schizophr. Bull..

[43] Burch GSJ, Steel C, Hemsley DR (1998). Oxford-liverpool inventory of feelings and experiences: Reliability in an experimental population. Br. J. Clin. Psychol..

[44] Kessler RC (2005). The World Health Organization adult ADHD self-report scale (ASRS): a short screening scale for use in the general population. Psychol. Med..

[45] Das D, Cherbuin N, Butterworth P, Anstey KJ, Easteal S (2012). A population-based study of attention deficit/hyperactivity disorder symptoms and associated impairment in middle-aged adults. PLoS ONE.

[46] Adler LA (2006). Validity of pilot adult ADHD self- report scale (ASRS) to rate adult ADHD symptoms. Ann. Clin. Psychiatry.

[47] Ritvo RA (2011). The ritvo autism asperger diagnostic scale-revised (RAADS-R): A scale to assist the diagnosis of autism spectrum disorder in adults: an international validation study. J. Autism Dev. Disord..

[48] Hahn E, Gottschling J, Spinath FM (2012). Short measurements of personality: Validity and reliability of the GSOEP big five inventory (BFI-S). J. Res. Personal..

[49] Rosseel Y (2012). lavaan: An R package for structural equation modeling. J. Stat. Softw..

[50] R Development Core Team. R: A language and environment for statistical computing. (2021).

[51] World Medical Association Declaration of Helsinki (2013). Ethical principles for medical research involving human subjects. JAMA.

[52] Bowes SM, Costello TH, Ma W, Lilienfeld SO (2021). Looking under the tinfoil hat: Clarifying the personological and psychopathological correlates of conspiracy beliefs. J. Pers..

[53] Goreis A, Voracek M (2019). A systematic review and meta-analysis of psychological research on conspiracy beliefs: Field characteristics, measurement instruments, and associations with personality traits. Front. Psychol..

[54] March E, Springer J (2019). Belief in conspiracy theories: The predictive role of schizotypy, machiavellianism, and primary psychopathy. PLoS ONE.

[55] Lindström M (2005). social capital, the miniaturization of community and high alcohol consumption: A population-based study. Alcohol. Alcohol..

[56] Lundborg P (2005). Social capital and substance use among Swedish adolescents: An explorative study. Soc. Sci. Med..

[57] Kotov R, Gamez W, Schmidt F, Watson D (2010). Linking, “big” personality traits to anxiety, depressive, and substance use disorders: A meta-analysis. Psychol. Bull..

[58] Bauman Z (2014). After Snowden: Rethinking the Impact of Surveillance. Int. Polit. Sociol..

[59] Maras M-H (2015). Internet of Things: Security and privacy implications. Int. Data Priv. Law.

[60] Barkun M (2013). A Culture of Conspiracy: Apocalyptic Visions in Contemporary AMERICA.

[61] Voss JL, Federmeier KD, Paller KA (2012). The potato chip really Does look like elvis! Neural hallmarks of conceptual processing associated with finding novel shapes subjectively meaningful. Cereb. Cortex.

[62] Poon K-T, Chen Z, Wong W-Y (2020). Beliefs in conspiracy theories following ostracism. Pers. Soc. Psychol. Bull..

[63] Strauss W, Howe N (1998). The Fourth Turning: An American Prophecy.

[64] *The science of self-report: implications for research and practice*. (Lawrence Erlbaum, 2000).

[65] Macleod J, Hickman M, Smith GD (2005). Reporting bias and self-reported drug use. Addict. Abingdon Engl..

