# Supplementary material for: Alternative beliefs in psychedelic drug users
Source: Sci Rep. 2023 Sep 30;13:16432. doi: 10.1038/s41598-023-42444-z (PMC10542757; doi:10.1038/s41598-023-42444-z)
Supplement: Supplementary file 1 — Supplementary Information. [file 41598_2023_42444_MOESM1_ESM.pdf]

Supplement

Alternative beliefs in psychedelic drug users

Authors:

Lebedev AV [1,2], Acar K [1,2], Horntvedt O [1], Carbera A [1], Simonsson O [3], Osika W [3], Ingvar M [1,2], Petrovic P [1,2]

Affiliations:

[1] Department of Clinical Neuroscience, Karolinska Institutet, Stockholm, Sweden

[2] Center for Cognitive and Computational Neuroscience, Karolinska Institutet, Stockholm, Sweden

[3] Department of Clinical Neuroscience, Center for Psychiatry Research (W.O.), Karolinska Institute, Sweden

Table S1

Conspiracy Mentality Questionnaire - Swedish

1. Det händer många viktiga saker i världen som allmänheten aldrig informeras om.
2. Politiker brukar inte berätta för oss de sanna motiven för sina beslut.
3. Statliga myndigheter övervakar alla medborgare noggrant.
4. Händelser som på ett ytligt plan verkar sakna koppling är ofta resultatet av hemliga aktiviteter.
5. Det finns hemliga organisationer som starkt påverkar politiska beslut.

Table S2

Epistemic Belief Scale – Swedish Version

**Faith in Intuition for facts:**	
Feel1	Jag litar på att min magkänsla säger mig vad som är sant eller falskt
Feel2	Jag litar på mitt första intryck av fakta
Feel3	Mitt första intryck är nästan alltid rätt
Feel4	Jag kan vanligtvis känna om ett påstående är rätt eller fel, även om jag inte kan förklara varför
**Need for evidence:**	
Evid1	Belägg är viktigare än huruvida någonting känns sant
Evid2	En intuition måste bekräftas med underlag
Evid3	Jag litar på att fakta, inte min intuition, avgör vad som är sant
Evid4	Jag måste kunna rättfärdiga mina övertygelser med belägg
**Truth is political:**	
Poli1	Fakta styrs av de som har makt
Poli2	Makt avgör vad som räknas som sanning
Poli3	Vetenskapliga slutsatser formas av politiken
Poli4	"Fakta" beror på dess politiska kontext

Svarsalternativen går från "*Stämmer inte alls*" till "*Stämmer helt*".

Table S3
Data Collection Sources

- Initial screening: Demographics + PDI + O-LIFE + Diagnoses + drug-use (mini)
- Follow-up 1: BFI-S + CMQ + Drug use (extended)
- Follow-up 2: study participants + social media posting: EBS
- Scanning participants from the experimental arm of the larger study on *delusion proneness*

Table S4
Drug use frequency and beliefs in alternative facts (CMQ)

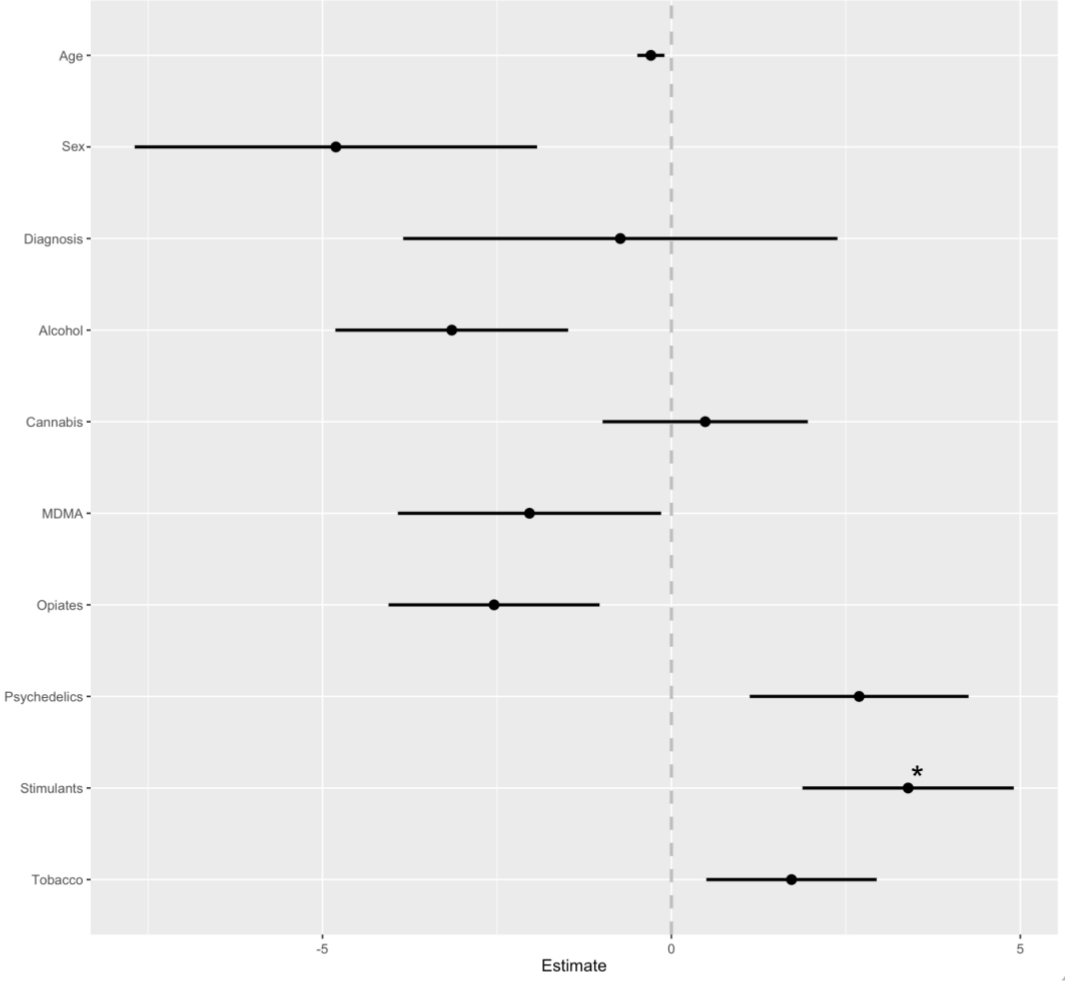

Estimate: non-standardized coefficients.

Table S5
Cross-correlations between study outcomes and psychiatric symptoms

Correlation Matrix for Scores on the Big Five Subscales, "Delusion-Proneness" and Recent Use of Drugs

		O	C	E	A	N	DP	Alcohol	Cannabis	MDMA	Opiates	Psychedelics	Stimulants	Tobacco
O	r	-												
C	r	.093	-											
E	r	.245***	.018	-										
A	r	.136**	.107*	.196***	-									
N	r	-.218***	-.130*	-.196***	-.097	-								
DP	r	.221***	-0.71	-.100	-.058	.240***	-							
Alcohol	r	.035	-.030	.124*	.006	-.057	-.082	-						
Cannabis	r	.230***	-.029	.041	-.162**	-.162**	.070	.180***	-					
MDMA	r	.178***	-.112*	.081	-.110*	-.110*	.134*	.092	.506***	-				
Opiates	r	-.030	-.053	-.015	.022	.022	-.027	.027	.247***	.249***	-			
Psychedelics	r	.248***	-.090	.061	-.130*	-.130*	.169**	.066	.470***	.645***	.210***	-		
Stimulants	r	.118*	-.120*	.074	-.038	-.038	.112	.177***	.427***	.606***	.376***	.424***	-	
Tobacco	r	.173***	.034	.088	-.100*	-.100*	.098	.225***	.452***	.266***	.252***	.239***	.396***	-

Note. N = 392. r = Pearson Correlation Coefficient. * = p < .05, ** p < .01, *** p < .001.

Correlation Matrix for Scores on the Big Five Subscales, Delusion Proneness and Frequent Use of Drugs

		O	C	E	A	N	DP	Alcohol	Cannabis	MDMA	Opiates	Psychedelics	Stimulants	Tobacco
O	r	-												
C	r	.093	-											
E	r	.245***	.018	-										
A	r	.136**	.107*	.196***	-									
N	r	-.218***	-.130*	-.196***	-.097	-								
DP	r	.221***	-0.71	-.100	-.058	.240***	-							
Alcohol	r	.084	-.040	.053	.010	-.090	-.060	-						
Cannabis	r	.221***	-.119*	.016	0.042	-.128*	.124*	.422***	-					
MDMA	r	.222***	-.107*	.064	.073	-.159**	.105	.225***	.622***	-				
Opiates	r	-.038	-.050	.008	.045	.006	-.021	.116*	.328***	.307***	-			
Psychedelics	r	.255***	-.139**	.002	.087	-.171***	.124*	.224***	.645***	.709***	.317***	-		
Stimulants	r	.085	-.091	.029	.093	-.038	.117	.257***	.599***	.701***	.504***	.581***	-	
Tobacco	r	.157**	-.003	.041	.063	-.116*	.101	.487***	.690***	.403***	.233***	.384***	.452***	-

Note. N = 392. r = Pearson Correlation Coefficient. * = p < .05, ** p < .01, *** p < .001. O = Openness, C = Conscientiousness, E = Extraversion, A = Agreeableness, N = Neuroticism, DP = Delusion Proneness.

Psychometric Analysis: CMQ

This study used a previously constructed, but unvalidated Swedish version of the CMQ. For this reason, a psychometric evaluation was performed in several different steps. All psychometric analyses were performed on the entire sample (N = 392). It consisted of 29% men, 41% women and 30% other/N/A, ages ranged between 15-67 years old. Comparison of the versions completed by English and Swedish speakers (provided at the end) concluded that strict invariance holds.

First, construct reliability (*CR*) was established (α =.837), which was in line with previously translated versions (Bruder et al., 2013). To evaluate structural validity, a confirmatory factor analysis (*CFA*) was performed. To evaluate goodness of fit, the

χ^2/degrees of freedom (*df*) ratio was used, with ≤ 5 indicating an acceptable fit, and ≤ 3 indicating a good fit. A nonsignificant value of χ^2 would indicate a failure to reject the null hypothesis that the hypothesized covariance matrix is identical to the observed covariance matrix, which usually is accepted as evidence of adequate fit (Schreiber et al., 2006). Moreover, cut-offs for estimates of goodness-of-fit were chosen according to general rules of thumbs (Hu & Bentler, 1999, Navarro & Foxcroft, 2019). A model was interpreted to have a satisfactory fit by the comparative fit index (*CFI*) > 0.9, the Tucker-Lewis fit index (*TLI*) > 0.9, the standardized root mean square residual (*SRMR*) < 0.09 and the root mean square error of approximation (*RMSEA*) between 0.05 to 0.10. A *CFI* > 0.95, *TLI* > 0.95, *SRMR* < 0.08, *RMSEA* < 0.05 and *PCLOSE* > .05 would indicate a good fit. According to (Hair, 2009), convergent validity can be explained by *CR* and Average Variance Extracted (*AVE*): convergent validity is observed when *CR* > *AVE*, and the *AVE* is > 0.5. Similarly, discriminant validity is confirmed when √(*AVE*) > largest correlation with other factors.

The data was treated as continuous variables. There were no missing data. All variables violated the normality assumption, as assessed by Shapiro-Wilk's test of normality. The null hypothesis was rejected for all tests (*p*-values < 0.001), suggesting a violation of multivariate normality. Box-cox transformations were performed in an attempt to normalize the distributions, without success. For this reason, weighted least squares was chosen as estimator.

All factor loadings were high and significant, with standardized coefficients ranging between 0.566 - 0.867. Examination of the *AVE* verified construct validity (*AVE* = 0.56). However, all fit parameters except the *SRMR* = .060, suggested a relatively bad fit (χ^2(5, *N* = 392) = 43.723, *p* < .001; *CFI* = .854; *TLI* = .707, *RMSEA* = .141 90%*CI*.104-.181, *PCLOSE* < .001).

After inspecting the modification indices, a second model was estimated, allowing several of the observed variables to correlate. A comparison showed that it fit the data significantly better than the first model (χ^2(5) = 40.071, *p* < .001) and model fit parameters indicated excellent fit (χ^2(0, *N* = 392) = 3.652, p < .001; *CFI* = .986; *TLI* = 1.000, *RMSEA* = .000 90%*CI*.000-.000, *PCLOSE* = N/A, *SRMR* = .017).

Even though the second model differs from the original, it does not contradict the theoretical background that underlies the construct *conspiracy mentality*". In fact, it has been shown that belief in one conspiracy theory is a strong predictor of endorsement of another conspiracy theory (Swami et al., 2013). In conclusion, initial findings indicate that construct

reliability and validity can be confirmed for a new, adapted version of the CMQ, however, further evaluation in other populations is needed, although this lies beyond the scope of this study.

Figure S6

A Path Diagram Representing the Factor Structure of the Conspiracy Mentality Questionnaire

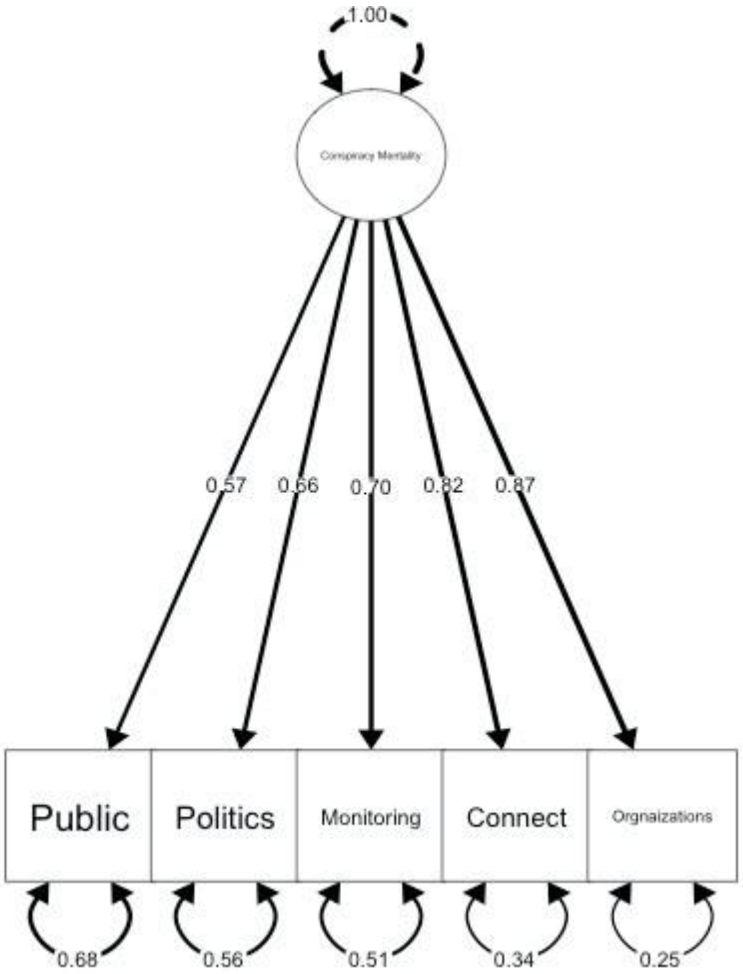

Note. $\chi^2(5, N = 392) = 43.723$, $p < .001$, *CFI* = .854, *TLI* = .707, *RMSEA* = .141 90%*CI*.104-.181, *PCLOSE* < .001, *SRMR* = .060

Figure S7

A Path Diagram Representing a Modification of the Factor Structure of the Conspiracy Mentality Questionnaire

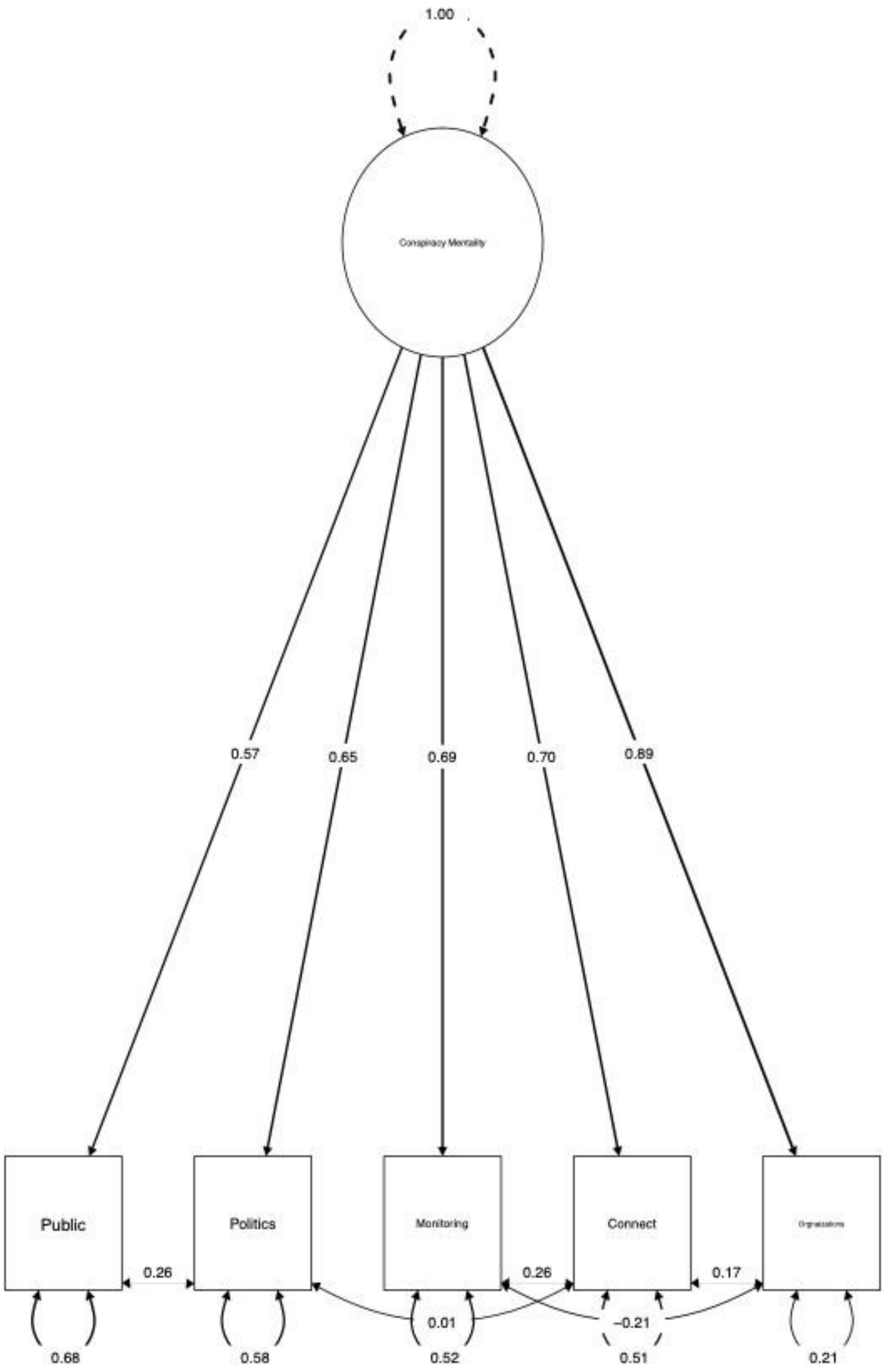

Note. $\chi^2(0, N = 392) = 3.652$, p < .001; *CFI* = .986; *TLI* = 1.000, *RMSEA* = .000 90%*CI*.000-.000, *PCLOSE* = N/A, *SRMR* = .017

Psychometric Analysis: EBS

As with the CMQ, there were no data on which subjects had used the Swedish version of the EBS. Therefore, all analyses were performed on the entire sample (N = 305), which consisted of 23% men, 40% women and 37% other/N/A, ages ranged between 18-67 years old.

Construct reliability was evaluated through scale reliability measures for the composite scales. It showed that the EBS maintained adequate internal consistency (α_{feel} = .764, α_{evid} = .789, α_{poli} = .841).

To evaluate structural validity a CFA was performed. The same cut-off values were used as previously mentioned. The data was treated as continuous variables. There were no missing data. All samples violated the normality assumption, as assessed by Shapiro-Wilk's test of normality (p-values < 0.05), indicating a violation of multivariate normality. Attempts to normalize the distributions using box-cox transformations were unsuccessful. For this reason, weighted least squares was chosen as estimator method.

The model showed significant and high factor loadings, with standardized coefficients ranging between 0.663-0.834. Examination of the *AVE* of each construct verified construct validity for all of the latent variables; *FI-facts* (*AVE* .517) *Need for evidence* (*AVE* .587) *Truth is political* (*AVE* 0.610). Moreover, discriminant validity was demonstrated by two ofthe latent variables (*Need for evidence*, *Truth is political*).

The model showed significant misfit (χ^2(51, N = 305) = 106.780, p < .001, but the χ^2/df ratio was good. The *RMSEA* = .060 90%*CI* .044-.076, *PCLOSE* = .145 and *SRMR* = .084) indicated that the model had a close fit, however *CFI* =.866 and *TLI* = .827 measures indicated that the model did not fit the data.

After inspecting the modification indices, a second model, where some of the observed variables were allowed to correlate, were computed. This model fit the data significantly better, than the model treating the observed variables as independent (χ^2(2)= 23.253, p < .001).
As the previous model, it showed significant misfit, (χ^2(83.528, N = 305) = 106.780, p = .002, although the χ^2/df was still good. All other fit parameters indicated a good fit (*CFI* = .917, *RMSEA* = .048 90%*CI* .030-.065, *PCLOSE* = .547 and *SRMR* = .076) except for the *TLI* = 0.888.

In sum, our analyses show that the EBS demonstrates construct reliability and validity and a modified model that goes well, with the underlying theory of the EBS shows a good fit with the data.

Figure S8

A Path Diagram Representing the Factor Structure of the Epistemic Belief Scale

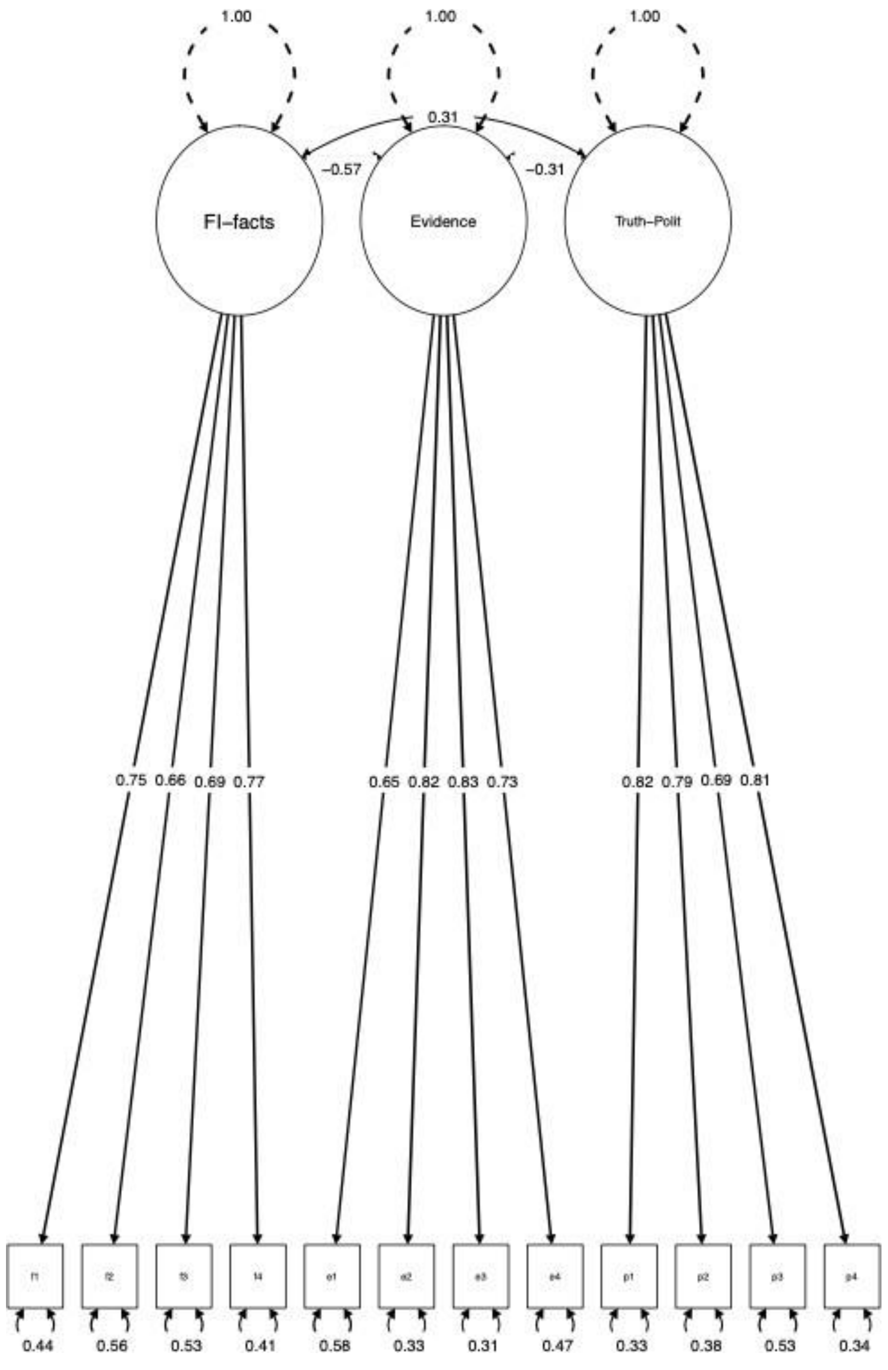

Note. $\chi^2(51, N = 305) = 106.780$, $p < .001$, *CFI* =.866 and *TLI* = .827, *RMSEA* = .060 90%*CI* .044-.076, *PCLOSE* = .145 and *SRMR* = .084

Figure S8

A Path Diagram Representing a Modification of the Factor Structure of the Epistemic BeliefScale

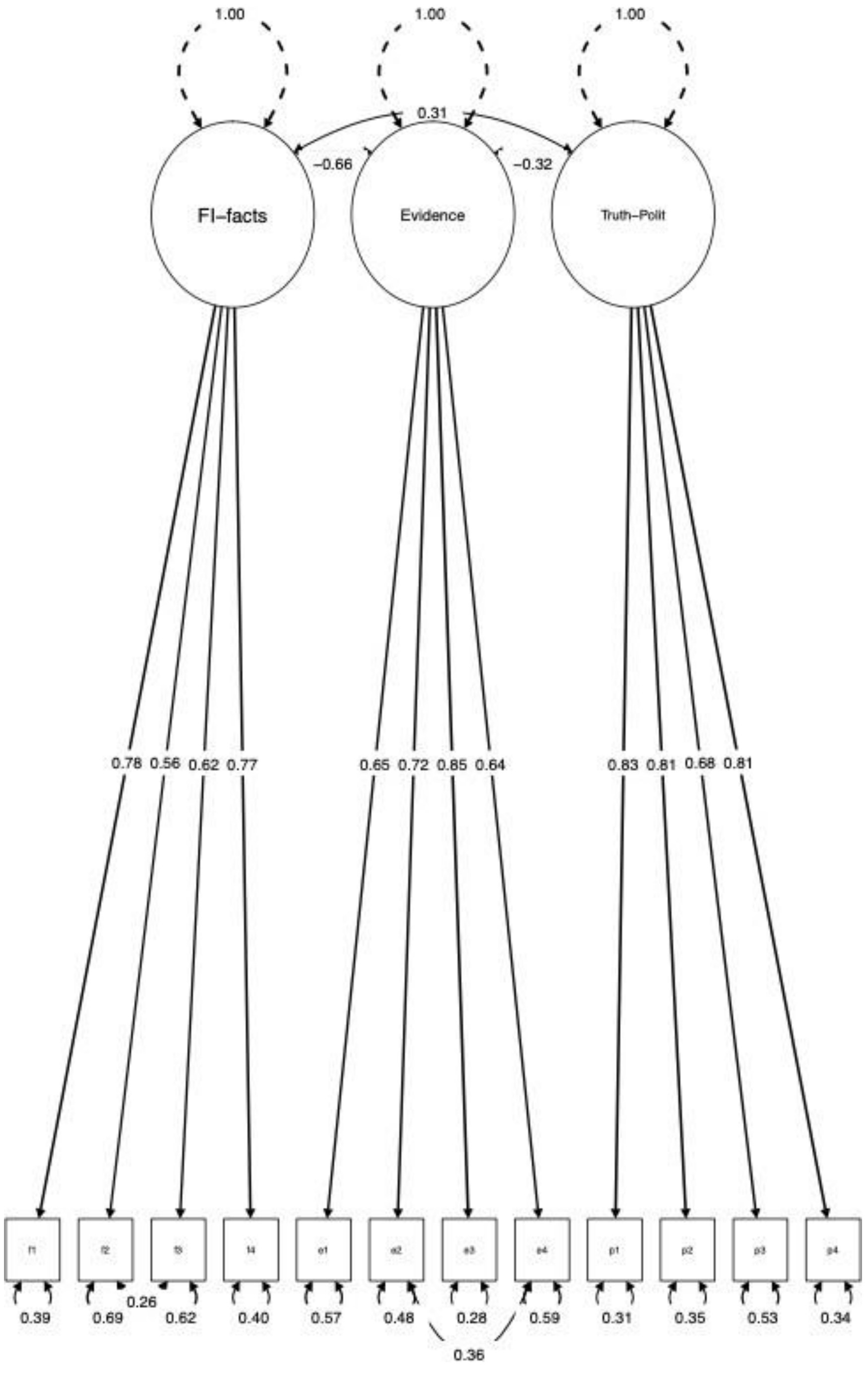

Note. χ^2(83.528, *N* = 305) = 106.780, *p* = .002, *CFI* = .917, *TLI* = 0.888, *RMSEA* = .048 90%*CI* .030-.065, *PCLOSE* = .547 and *SRMR* = .076.

Conspiracy Mentality Questionnaire (CMQ): Invariance Testing Results

Fit	DF	AIC	BIC	χ^2	χ^2Diff	RMSEA	DF-diff	Pr(>χ^2)
**Configural**	10	13877	13988	55.18	-	-	-	-
**Metric**	14	13873	13969	59.23	4.05	0.009	4	0.4
**Scalar**	18	13875	13957	69.45	10.22	0.1	4	0.037
**Strict**	23	13872	13935	76.55	7.1	0.05	5	0.21

Scalar invariance assumption was formally not met for English- and Swedish-speaking groups as indicated by significant (not corrected for multiple testing) χ^2 difference.
It can be concluded that at least metric invariance is met for CMQ.
Configural: fixed and free factor loadings (baseline model)
Metric (Invariance): factor loadings are constrained to be equal across groups to see if the scales of the factors are equivalent.
Scalar (Invariance): Both the factor loadings and the intercepts are constrained to be equal across groups, testing whether the origins of the scales are equivalent.
Strict (Invariance): The factor loadings, intercepts, and residuals are constrained to be equal across groups. The strongest form of invariance.

Epistemic Belief Scale (EBS): Invariance Testing Results

Fit	DF	AIC	BIC	χ^2	χ^2Diff	RMSEA	DF-diff	Pr(>χ^2)
**Configural**	102	10031	10321	211.81	-	-	-	-
**Metric**	111	10020	10277	219.47	7.66	<0.001	9	0.57
**Scalar**	120	10011	10234	227.85	8.38	<0.001	9	0.50
**Strict**	132	10000	10179	241.23	13.38	<0.027	12	0.34

None of the models resulted in significant χ^2 drop concluding that strict invariance assumption holds when evaluating English- and Swedish-speaking groups.

Configural: fixed and free factor loadings (baseline model)
Metric (Invariance): factor loadings are constrained to be equal across groups to see if the scales of the factors are equivalent.
Scalar (Invariance): Both the factor loadings and the intercepts are constrained to be equal across groups, testing whether the origins of the scales are equivalent.
Strict (Invariance): The factor loadings, intercepts, and residuals are constrained to be equal across groups. The strongest form of invariance.

Difference in CMQ scores between Swedish and Non-Swedish samples:

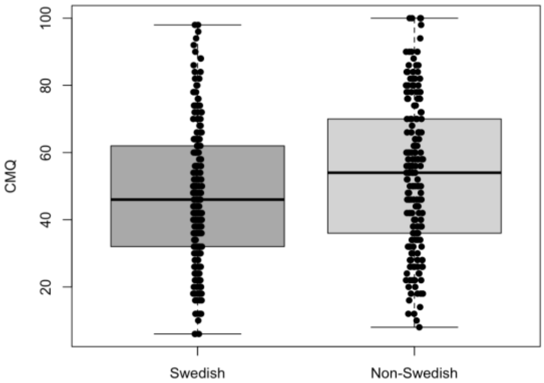

Swedish sample scored significantly lower on CMQ compared to the non-Swedish sample (47.18 ±20.98 and 53.08±22, respectively; t_{382}=2.71, p=0.007), with largest difference observed in items “Monitoring” (p_{corr}=0.0026) and “Hidden connections” (p_{corr}=0.041).

EBS scores were equivalent between the groups (see below).

Difference in all scale items (Non-Swedish vs Swedish):

CMQ:

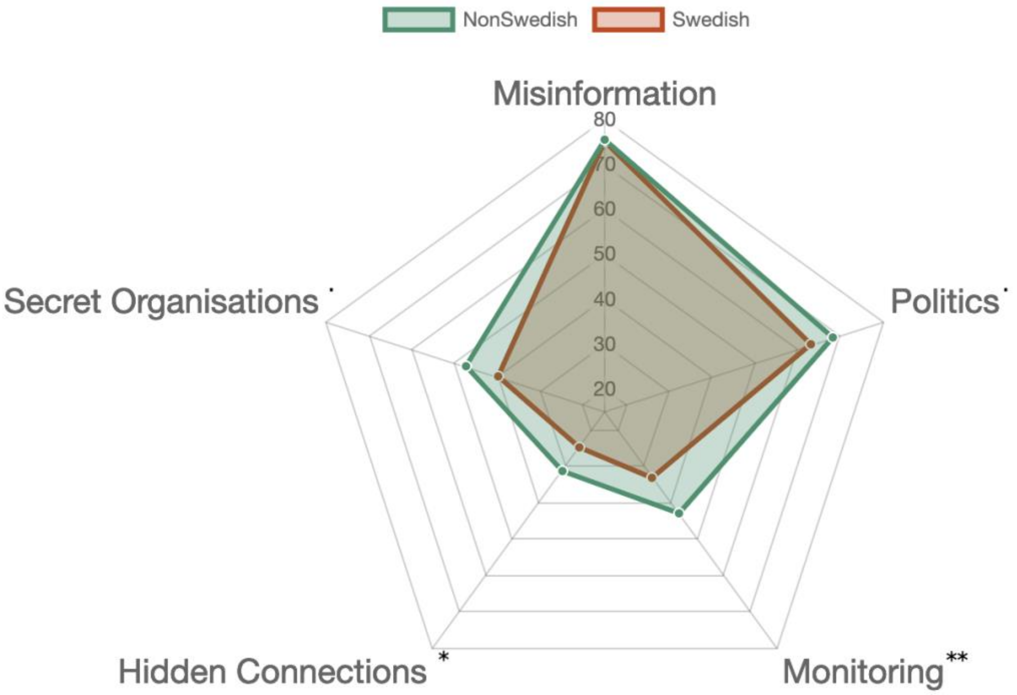

. – p_{corr} <- 0.1; * – p_{corr} <- 0.05; ** – p_{corr} <- 0.01

EBS:

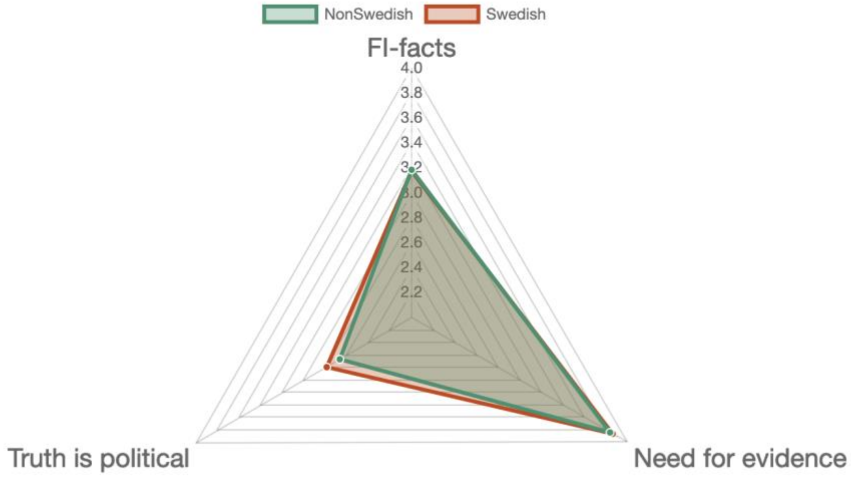

Coefficient Plot of Multiple Regression Models for the Predictors of Mean Score on the CMQ: Non-Swedish.

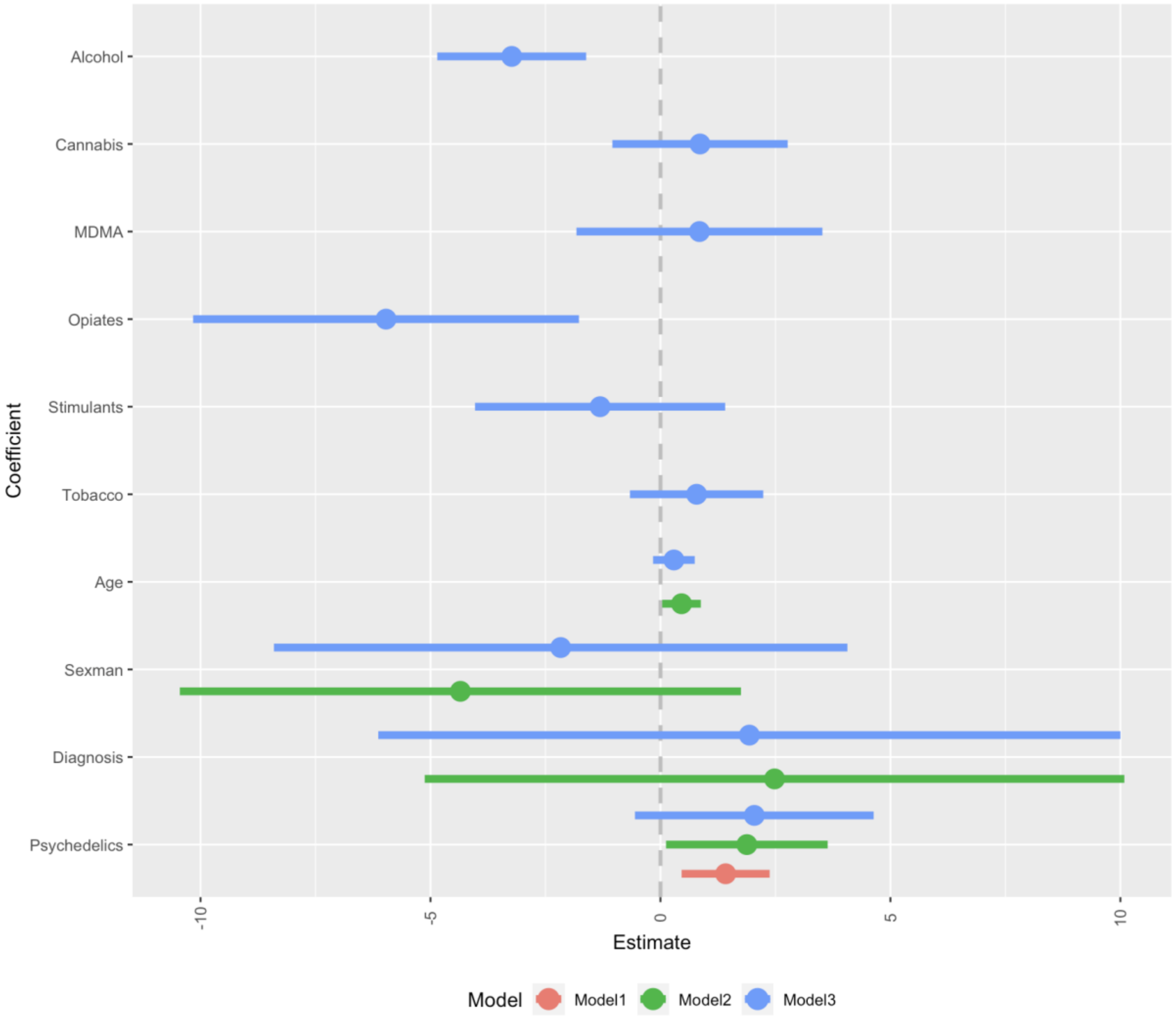

Model 1: minimal (psychedelic use and intercept), Model 2: demographic adjustment, Model 3: full model adjusted for concomitant drug use. Error bars denote 95% confidence intervals.

Coefficient Plot of Multiple Regression Models for the Predictors of Mean Score on the CMQ: Swedish.

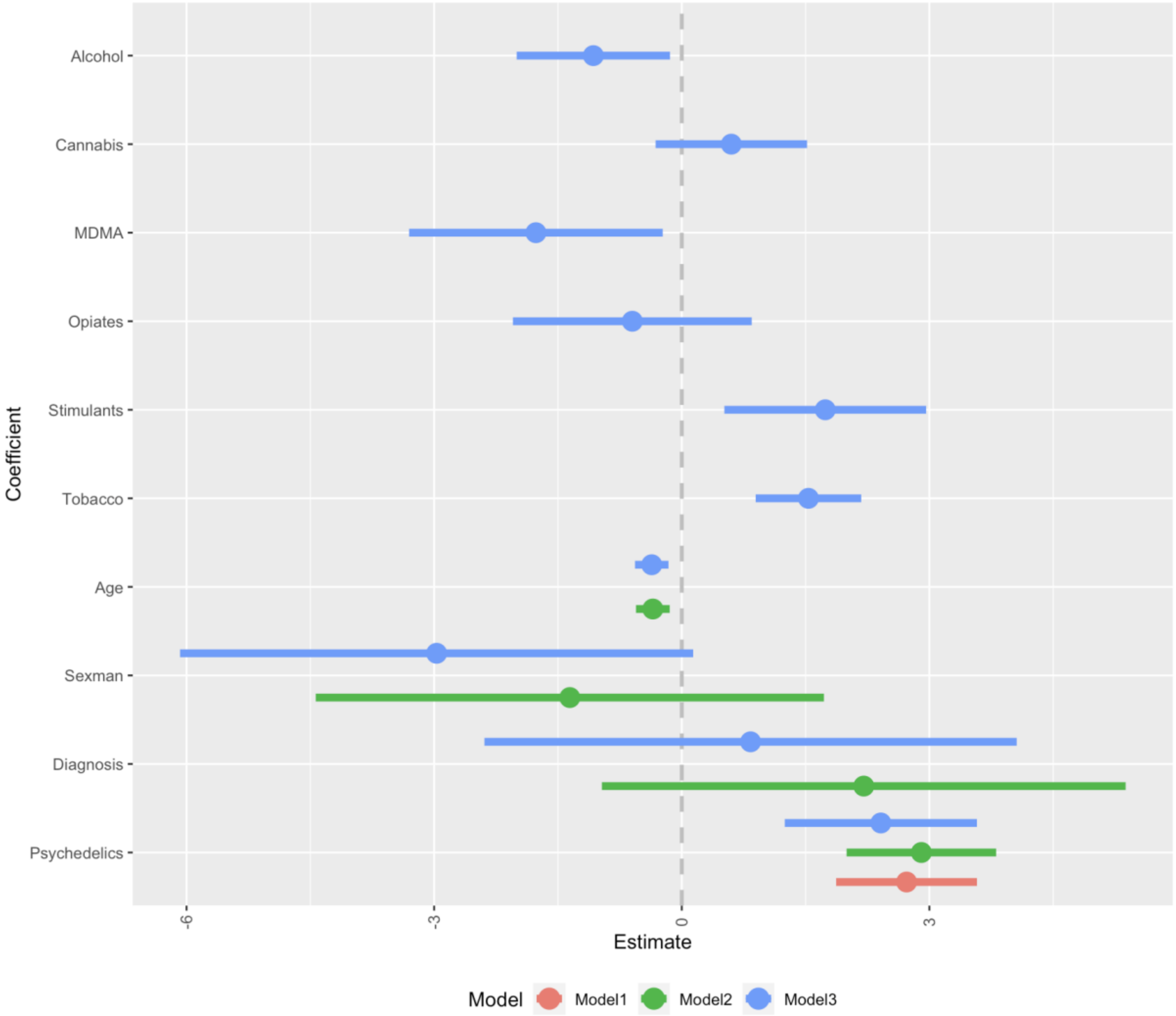

Model 1: minimal (psychedelic use and intercept), Model 2: demographic adjustment, Model 3: full model adjusted for concomitant drug use. Error bars denote 95% confidence intervals.

Coefficient Plot of Multiple Regression Models for the Predictors of the EBS score: Truth Is Political: Non-Swedish.

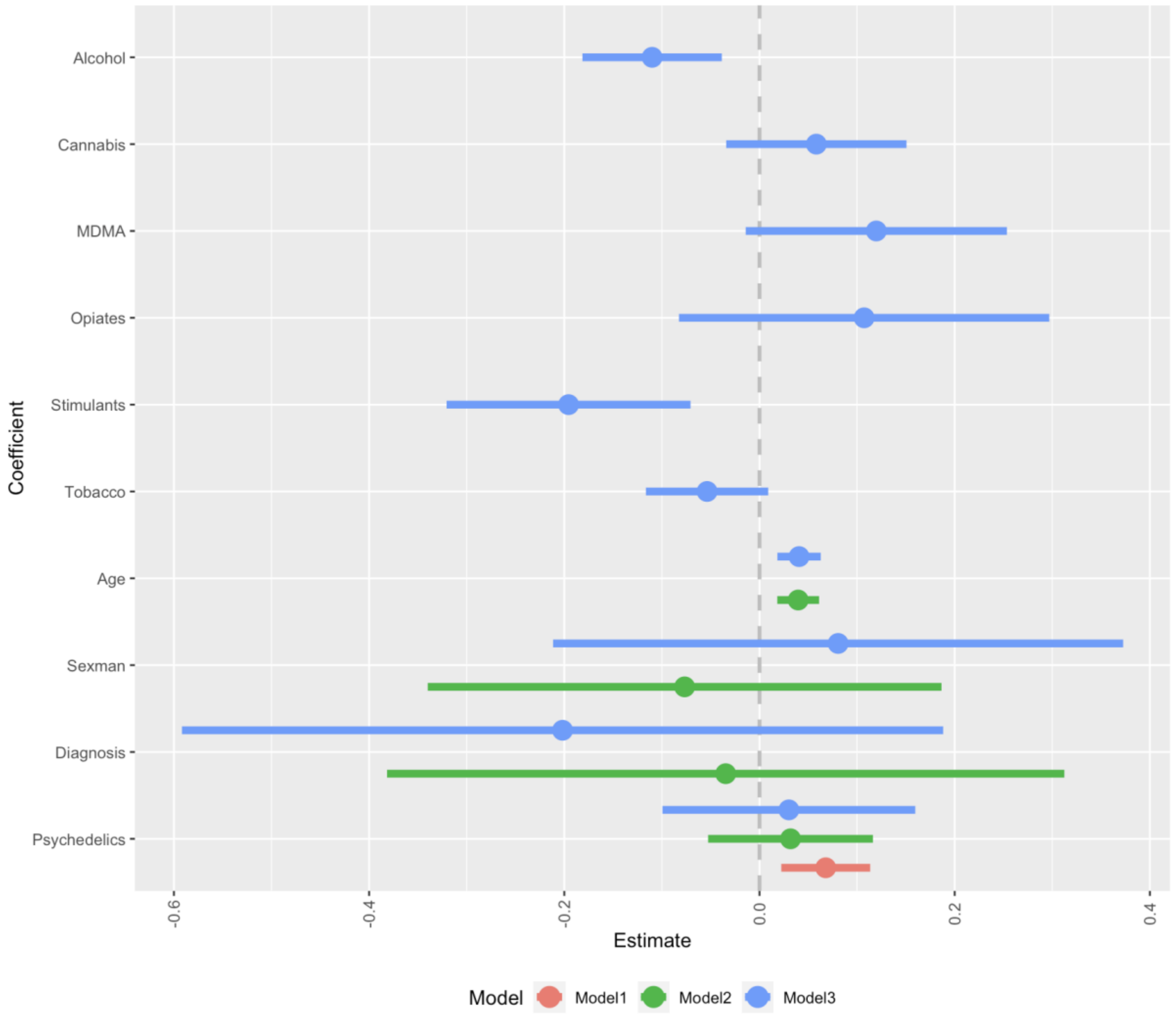

Model 1: minimal (psychedelic use and intercept), Model 2: demographic adjustment, Model 3: full model adjusted for concomitant drug use. Error bars denote 95% confidence intervals.

Coefficient Plot of Multiple Regression Models for the Predictors of the EBS score: Truth Is Political: Swedish.

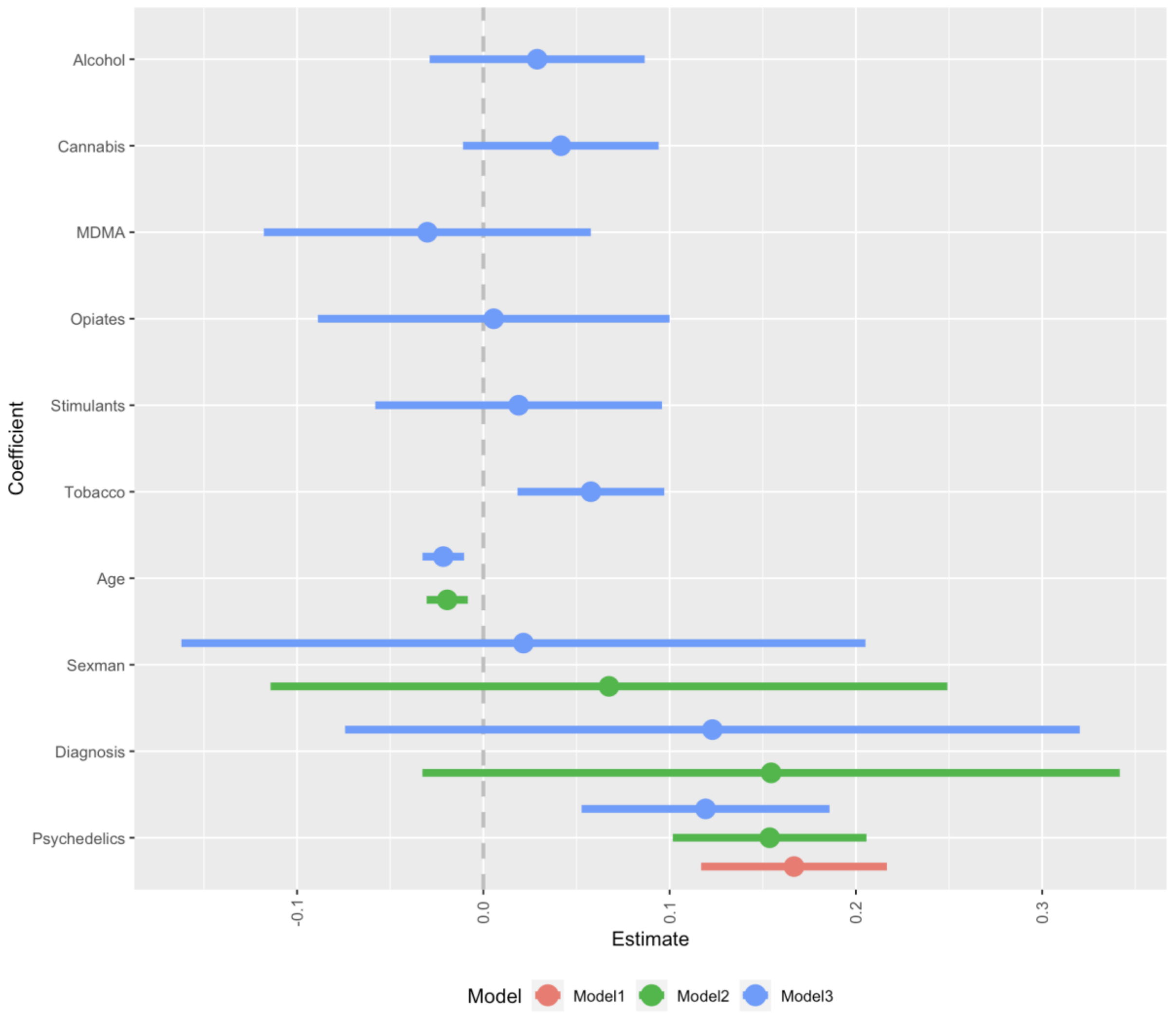

Model 1: minimal (psychedelic use and intercept), Model 2: demographic adjustment, Model 3: full model adjusted for concomitant drug use. Error bars denote 95% confidence intervals.
